# Estimating vaccine coverage in conflict settings using geospatial methods: a case study in Borno state, Nigeria

**DOI:** 10.1038/s41598-023-37947-8

**Published:** 2023-07-08

**Authors:** Alyssa N. Sbarra, Sam Rolfe, Emily Haeuser, Jason Q. Nguyen, Aishatu Adamu, Daniel Adeyinka, Olufemi Ajumobi, Chisom Akunna, Ganiyu Amusa, Tukur Dahiru, Michael Ekholuenetale, Christopher Esezobor, Kayode Fowobaje, Simon I. Hay, Charles Ibeneme, Segun Emmanuel Ibitoye, Olayinka Ilesanmi, Gbenga Kayode, Kris Krohn, Stephen S. Lim, Lyla E. Medeiros, Shafiu Mohammed, Vincent Nwatah, Anselm Okoro, Andrew T. Olagunju, Bolajoko O. Olusanya, Osayomwanbo Osarenotor, Mayowa Owolabi, Brandon Pickering, Mu’awiyyah Babale Sufiyan, Benjamin Uzochukwu, Ally Walker, Jonathan F. Mosser

**Affiliations:** 1 Institute for Health Metrics and Evaluation, University of Washington, 3980 15th Ave NE, Seattle, WA 98105 USA; 2 Community Medicine Department, Bayero University Kano, Kano, Nigeria; 3 Department of Infectious Disease Epidemiology, London School of Hygiene and Tropical Medicine, London, UK; 4 Department of Community Health and Epidemiology, University of Saskatchewan, Saskatoon, Canada; 5 Department of Public Health, Federal Ministry of Health, Abuja, Nigeria; 6 School of Public Health, University of Nevada Reno, Reno, USA; 7 National Malaria Elimination Program, Federal Ministry of Health, Abuja, Nigeria; 8 Department of Public Health, The Intercountry Centre for Oral Health (ICOH) for Africa, Jos, Nigeria; 9 Department of Medicine, University of Jos, Jos, Nigeria; 10 Department of Internal Medicine, Jos University Teaching Hospital, Jos, Nigeria; 11 Department of Community Medicine, Ahmadu Bello University, Zaria, Nigeria; 12 Department of Epidemiology and Medical Statistics, University of Ibadan, Ibadan, Nigeria; 13 Faculty of Public Health, University of Ibadan, Ibadan, Nigeria; 14 Department of Paediatrics, University of Lagos, Lagos, Nigeria; 15 Department of Paediatrics, Lagos University Teaching Hospital, Lagos, Nigeria; 16 Child Survival Unit, Centre for African Newborn Health and Nutrition, Ibadan, Nigeria; 17 Department of Health Metrics Sciences, University of Washington, Seattle, USA; 18 Department of Public Health and Disease Control, Abia State Ministry of Health, Umuahia, Nigeria; 19 Nigerian Field Epidemiology and Laboratory Training Program, African Field Epidemiology Network, Abuja, Nigeria; 20 Department of Health Promotion and Education, University of Ibadan, Ibadan, Nigeria; 21 Department of Community Medicine, University of Ibadan, Ibadan, Nigeria; 22 Department of Community Medicine, University College Hospital, Ibadan, Nigeria; 23 International Research Center of Excellence, Institute of Human Virology Nigeria, Abuja, Nigeria; 24 Julius Centre for Health Sciences and Primary Care, Utrecht University, Utrecht, The Netherlands; 25 Health Systems and Policy Research Unit, Ahmadu Bello University, Zaria, Nigeria; 26 Department of Healthcare Management, Technical University of Berlin, Berlin, Germany; 27 Department of Pediatrics, National Hospital Abuja, Abuja, Nigeria; 28 Department of International Public Health, University of Liverpool, Liverpool, UK; 29 Society for Family Health, Abuja, Nigeria; 30 Department of Psychiatry and Behavioural Neurosciences, McMaster University, Hamilton, Canada; 31 Department of Psychiatry, University of Lagos, Lagos, Nigeria; 32 Centre for Healthy Start Initiative, Ikoyi, Lagos, Nigeria; 33 Department of Environmental Management and Toxicology, University of Benin, Benin City, Nigeria; 34 Department of Medicine, University of Ibadan, Ibadan, Nigeria; 35 Department of Medicine, University College Hospital, Ibadan, Nigeria; 36 Department of Community Medicine, University of Nigeria Nsukka, Nsukka, Nigeria

**Keywords:** Epidemiology, Health services

## Abstract

Reliable estimates of subnational vaccination coverage are critical to track progress towards global immunisation targets and ensure equitable health outcomes for all children. However, conflict can limit the reliability of coverage estimates from traditional household-based surveys due to an inability to sample in unsafe and insecure areas and increased uncertainty in underlying population estimates. In these situations, model-based geostatistical (MBG) approaches offer alternative coverage estimates for administrative units affected by conflict. We estimated first- and third-dose diphtheria-tetanus-pertussis vaccine coverage in Borno state, Nigeria, using a spatiotemporal MBG modelling approach, then compared these to estimates from recent conflict-affected, household-based surveys. We compared sampling cluster locations from recent household-based surveys to geolocated data on conflict locations and modelled spatial coverage estimates, while also investigating the importance of reliable population estimates when assessing coverage in conflict settings. These results demonstrate that geospatially-modelled coverage estimates can be a valuable additional tool to understand coverage in locations where conflict prevents representative sampling.

## Introduction

Vaccination coverage in Nigeria has been estimated nationally and subnationally through numerous efforts, including via surveys, administrative data, and various modelling approaches^1,2^. While national-level coverage of at least three doses of the diphtheria-tetanus-pertussis (DTP3) vaccine has increased from an estimated 26% in 2000 to 60% in 2019, substantial subnational heterogeneity in coverage remains, with coverage estimates generally higher in the south than in the north^1,3^. Ongoing conflict, terrorism, and political instability persisting in northern and north-eastern portions of the country in states such as Borno, Yobe, Adamawa, and Taraba further widen the disparities between these regions. In these states, particularly Borno, the ongoing activities of the Boko Haram terrorist group present additional barriers to reaching children with vaccination programs^4,5^.

In conflict settings like Borno state, both vaccine delivery and the ability to reliably monitor vaccine coverage via household surveys can be compromised^6,7^. The Equity Reference Group for Immunization emphasises the importance of understanding vaccination coverage in conflict settings, while acknowledging that assessing immunisation status in these settings is extremely challenging^8^. For safety and security reasons, surveys may not be able to sample from subnational geographies where active conflict is present. This leaves vulnerable populations of unvaccinated children unrepresented in both national and state-level report estimates and underlying survey microdata^9^. In Borno state, for example, recent household-based surveys were only able to sample from a subset of planned clusters, noting in reports that results were therefore not representative of the entire state^9,10^.

Leveraging complex relationships of outcomes and covariates across space and time, model-based geostatistical (MBG) methods can provide alternative vaccine coverage estimates for areas where data are under-representative or unavailable. MBG methods have been previously used to estimate a wide variety of health-related outcomes and interventions, including DTP coverage^1,11–13^. DTP coverage is often used to measure the performance of routine childhood vaccine delivery programs from the initial encounter (i.e., DTP1 coverage) to the completion of the primary infant series (i.e., DTP3 coverage)^14^. In this work, we assess the use of geostatistical models to estimate vaccination coverage in conflict settings using the example of Borno state, Nigeria and consider the use of model-based estimates alongside survey estimates as an alternative tool for assessing coverage in such settings.

## Results

A significant proportion of conflict within Nigeria has been documented in the most north-eastern regions, including Borno state. In years 2016 through 2018, there were 1044 conflict-related incidents reported in Borno state alone, with a total of 7533 fatalities in all three years (Supplementary Fig. 1). These conflicts, in Borno state only, constitute 20.6% of all conflict-related incidents across Nigeria from 2016 to 2018. Conflict-related incidents were recorded in 24 of 27 local government areas (LGAs) in Borno state in 2016, 25 in 2017, and 23 in 2018.

**Figure 1:**
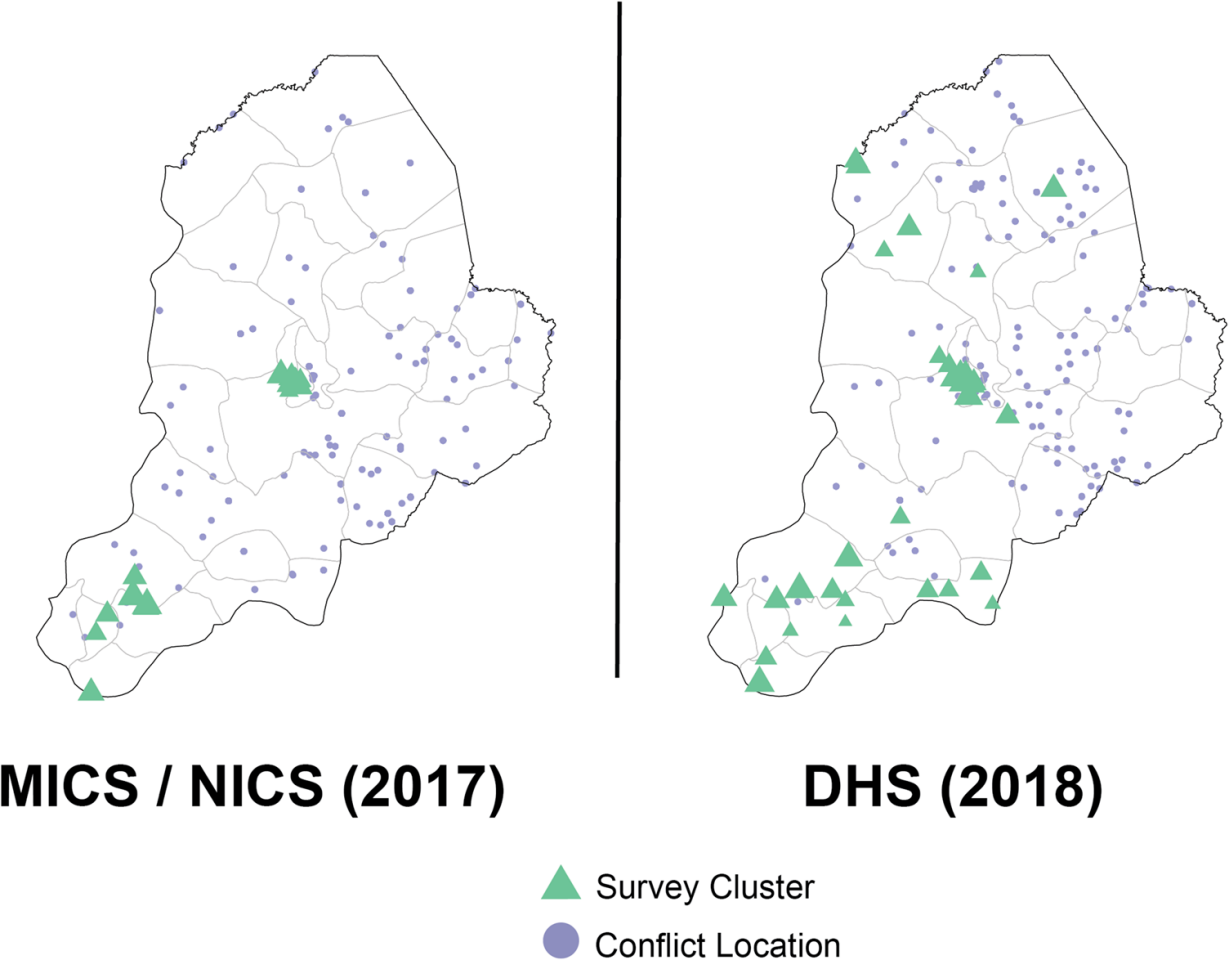
Locations of conflict and survey data collection within Borno state. For both the 2016–17 Multiple Indicator Cluster Survey (MICS/NICS) and 2018 Demographic and Health Survey (DHS), the location of each cluster sample during data collection is shown in green triangles, for the DHS sized by number of children sampled. Purple circles show the location of all types of conflict data points as part of the Armed Conflict Location & Event Data Project (ACLED) database in each survey year.

Both recent DHS and MICS/NICS surveys indicate that Borno estimates are not representative at the state level due to sampling limitations associated with insecurity. Notably, of the 27 LGAs in Borno state, the 2016–17 MICS/NICS only sampled in six and the 2018 DHS only sampled in 14. Five of six sampled LGAs in the 2016–17 MICS/NICS were experiencing conflict in 2017; similarly, 12 of 14 LGAs sampled by the 2018 DHS were also experiencing conflict, as reported by ACLED in 2018 (Fig. 1). From the three geo-located surveys with data available more granular than the state level in Nigeria since 2010, 8/27 (29.6%) of LGAs have not been sampled in Borno state, compared to 29/774 (3.7%) in all of Nigeria.

Geospatial population estimates suggest that a substantial proportion of children under 5 years of age in Borno state live in LGAs that were not represented in the MICS/NICS or DHS survey samples. Sampled LGAs from the 2016–17 MICS/NICS contain 26.6% of the under-5 population in Borno state according to WorldPop population estimates and 31.1% according to GRID3 estimates. Similarly, the 2018 DHS sampled LGAs containing 59.8% of the underlying population according to WorldPop estimates and 65.1% according to GRID3.

Per modelled estimates, DTP1 coverage increased in 98.1% (95% uncertainty interval (UI): 88.9–100%) of LGAs (N = 27) in Borno state from 2000 to 2019. These increases mirror broad gains across all of Nigeria, as DTP1 coverage increased in 95.1% (95% UI: 90.0–98.8%) of all LGAs (N = 774) between 2000 and 2019. In Borno state, DTP1 coverage increased from 21.3% (95% UI: 16.7–26.6%) in 2000 to 51.9% (95% UI: 41.6–63.8%) in 2019, while DTP3 coverage increased from 10.9% (95% UI: 7.3–15.5%) to 40.0% (95% UI: 28.6–53.3%). State-level coverage estimates in Borno did not change substantially when LGA-level population weights from GRID3 were used for aggregation instead of those from WorldPop (2019 GRID3-based DTP3 coverage: 41.6%, DTP1 coverage: 52.5%). Modelled estimates in Borno state suggest, however, substantially lower coverage for both DTP1 and DTP3 than reported by the most recent available surveys (Table 1). This pattern was more consistently observed in Borno state than in other states with representative sampling (Supplementary Figs. 2–5).

**Table 1:** Comparison of survey reported and spatiotemporal model-based geostatistical estimates of first- and third-dose diphtheria-tetanus-pertussis vaccination (DTP1 and DTP3) coverage in Borno state.

	2016–17 MICS/NICS		2018 DHS	
	2016 DTP1 coverage	2016 DTP3 coverage	2017 DTP1 coverage	2017 DTP3 coverage
Reported estimate*	72.9%	47.7%	56.2%	36.0% (95% CI: 27.3–44.7%)
MBG estimate	38.4% (95% UI: 33.2–43.7%)	27.0% (95% UI: 22.0–32.2%)	41.3% (95% UI: 35.7–47.3%)	27.5% (95% UI: 21.1–33.6%)
Notes regarding sampling	Survey report contained information on 101 enumeration areas which could not be sampled due to security inaccessibility especially in Borno and Yobe states.		11 survey clusters were dropped across the study, including 1 in Borno state. Also, 11 LGAs (containing 39% of Borno state residents) were dropped and data collection took longer than expected due to security concerns.	

First- and third-dose diphtheria-tetanus-pertussis (DTP) coverage as reported from final 2016–17 Multiple Indicators Cluster Survey with National Immunization Coverage Survey supplement (MICS/NICS) and 2018 Demographic and Health Survey (DHS) reports in Borno state. 95% confidence interval (CI) of survey estimate was only provided for DTP3 coverage from the 2018 DHS report. Spatiotemporal model-based geostatistical (MBG) estimates of DTP coverage in the same year are also shown.

*As noted in reports, these estimates are not representative for the entire state.

**Figure 2:**
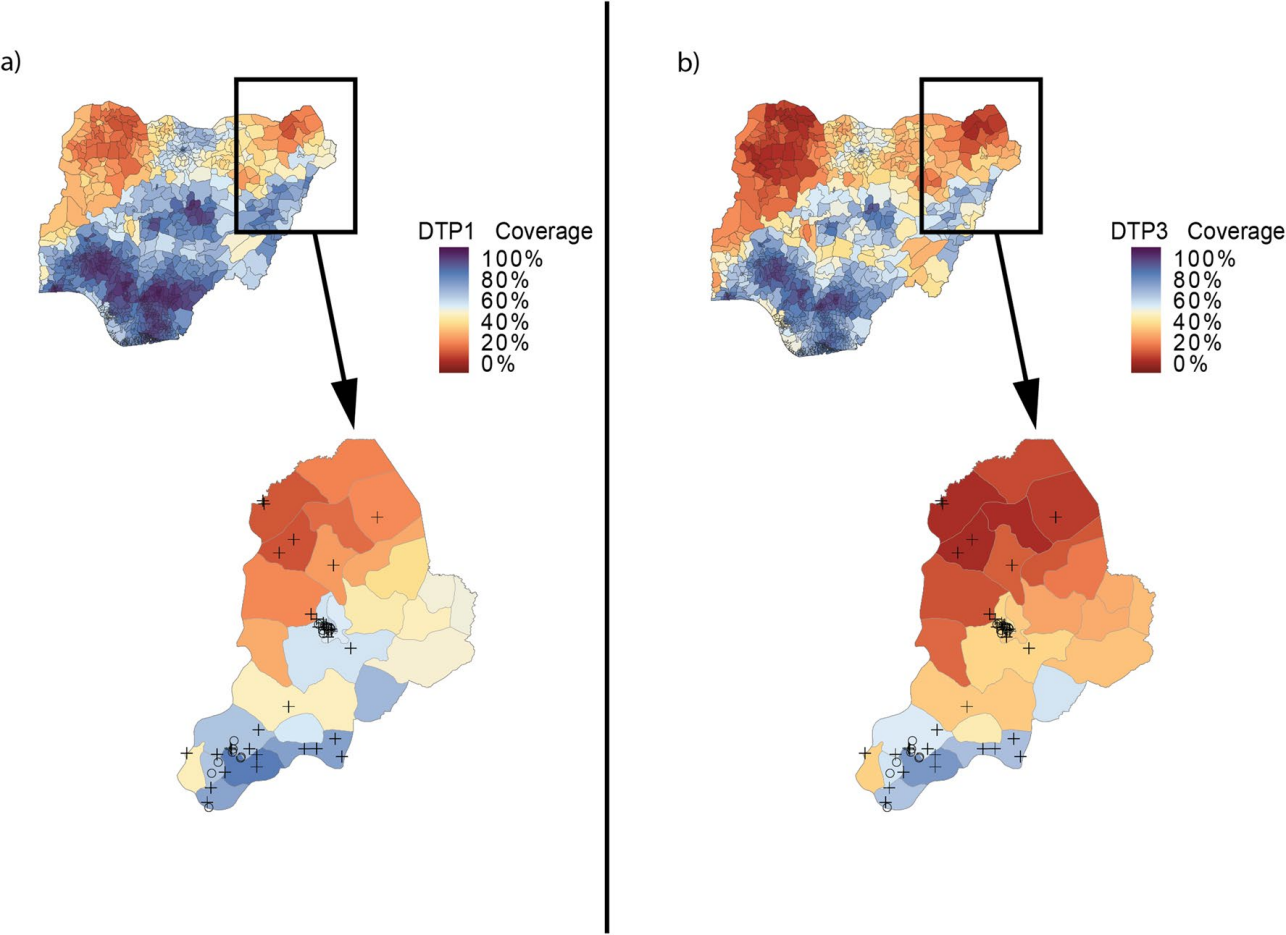
DTP1 and DTP3 coverage in Nigeria in 2018 with survey cluster locations in Borno state. Local governing authority (LGA) level coverage of DTP1 (**a**) and DTP3 (**b**) in Nigeria in 2018, aggregated from 5 × 5-km^2^ geospatial modelled estimates using second administrative units from the Database of Global Administrative Areas. In Borno state inset, locations of MICS/NICS sampling clusters (open circles) and DHS clusters ( +) are shown.

**Figure 3:**
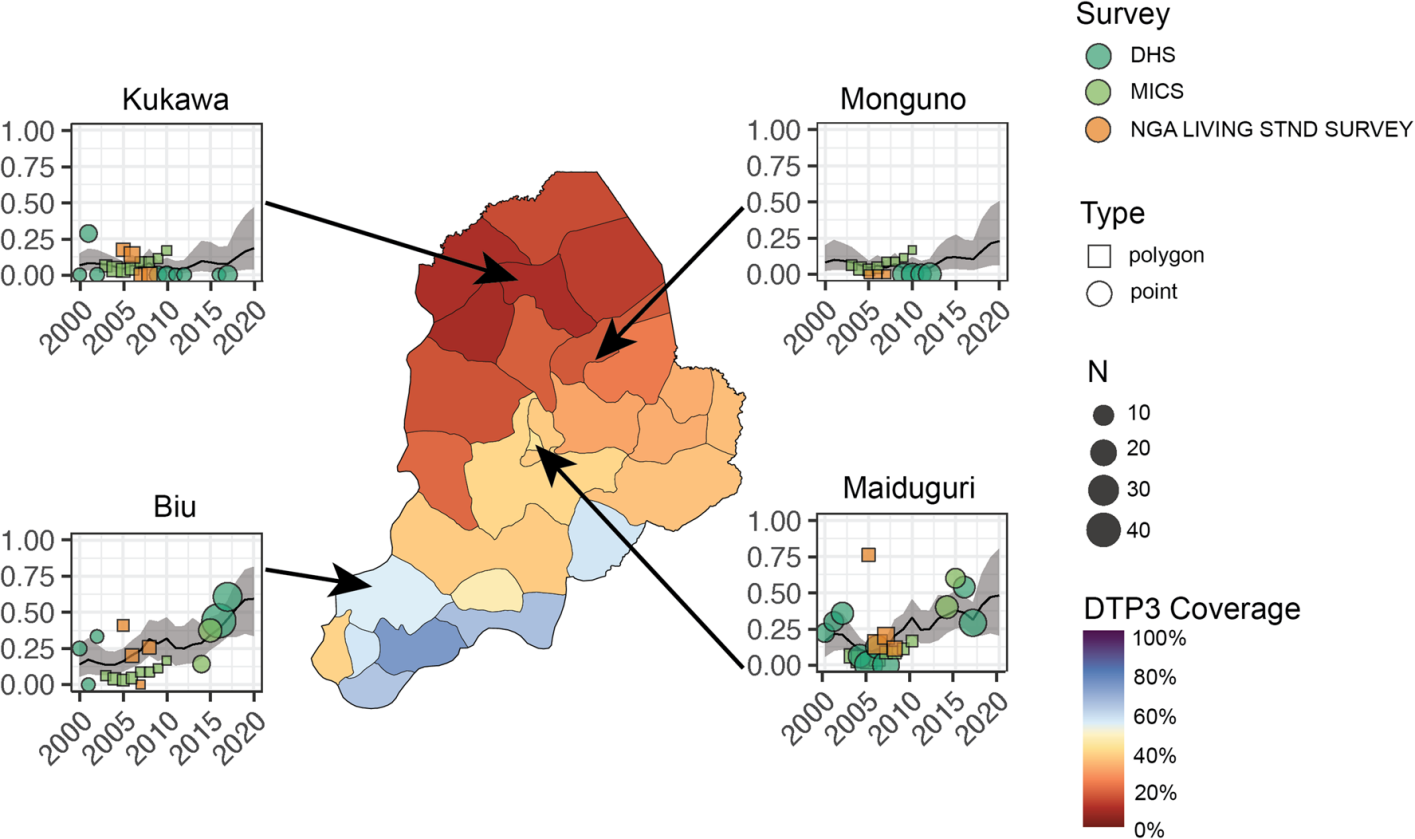
Borno state LGA-level time series of DTP3 coverage. Local governing authority (LGA) level estimates in Borno state for DTP3 coverage in 2018. A time series for select LGAs is shown with data sources included in the model aggregated to the LGA unit for years 2000 to 2018.

Survey sampling was more frequent in the Maiduguri LGA and surrounding areas as well as southern Borno LGAs, such as Konduga and Biu, where modelled DTP3 and DTP1 coverage estimates tend to be higher (Fig. 2). The 2018 DHS sampled four LGAs in the northernmost part of Borno, such as Kukawa; clusters from these areas suggest extremely low coverage. In LGAs sampled during recent surveys, such as Biu, Maiduguri, and Kukawa, modelled estimates mirrored available survey data. In LGAs that have not been sampled in recent surveys, the geospatial model produced estimates of coverage using data from nearby locations, historical trends, and covariate patterns. In unsampled northern LGAs such as Monguno, clusters from nearby sampled LGAs (i.e., Kukawa) and historical trends suggest low coverage. The geospatial model consequently estimated low DTP3 coverage in Monguno (and similar LGAs) in 2018 (Fig. 3). Because the geospatial model estimates low coverage in most unsampled LGAs, aggregated Borno state-level modelled coverage estimates are lower than non-representative estimates from recent survey reports, which exclude unsampled LGAs.

## Discussion

Many factors could disrupt the representativeness of survey sampling when assessing vaccine coverage, including sampling bias, insufficient resources, and infrastructure challenges to reaching remote areas^15^. Conflict can also impede reliable data collection efforts, including household surveys, as well as severely disrupt routine vaccination programs, highlighting the importance of accurate measurement of coverage in these vulnerable communities^5,16,17^. Due to an inability to sample in conflict-affected areas, current survey-based estimates are understandably not representative for all LGAs in Borno state, Nigeria, and therefore provide an incomplete picture of the status of DTP1 and DTP3 coverage. Model-based geostatistical estimates infer coverage in unsampled areas using historical trends, nearby data, and spatial covariates, which provide an additional tool when investigating coverage in conflict-affected areas.

Geospatially modelled coverage estimates in Borno state are lower than the non-representative estimates reported by the most recent DHS and MICS/NICS surveys. Sampled locations from recent surveys in Borno state tend to be in locations with higher MBG-estimated coverage and higher population estimates. In places where conflict-related insecurity limits survey sampling, the resulting non-representative survey estimates must be interpreted with caution if examined alone. Both WorldPop and GRID3 estimate that a substantial proportion of the total population in Borno lives in LGAs that have not been sampled in the most recent DHS and MICS/NICS. However, there is documented evidence of substantial internal displacement in conflict-affected areas of Borno state^18^, which has persisted over time. Given the challenges associated with assessing population in conflict areas, population distribution estimates in the region – which contribute to our understanding of state-level vaccine coverage both from survey data and geospatial models – should be interpreted with caution.

Substantial conflict-associated service disruptions in Borno state have been well described^19–22^, suggesting that the lower estimates from geospatial modelling – driven by low estimated coverage in unsampled LGAs – are plausible, particularly as conflict, low coverage, and sampling limitations are likely to be highly correlated. These differences in coverage between modelled estimates and from survey reports have notable implications for monitoring and tracking progress. Globally comparable estimates across space and time are critical for understanding geographies lagging in progress to avoid leaving vulnerable children behind.

Broadly, communities affected by conflict may also be more likely to suffer long-term health impacts of lower vaccine coverage due to the many overlapping adverse effects of conflict on health. Low levels of vaccine-derived immunity can lead to accumulation of unvaccinated or partially vaccinated children and adults, resulting in ongoing disease transmission and risk for large outbreaks. For example, substantial diphtheria outbreaks have been noted in the Borno area^23^ , and polio eradication efforts in the region have been threatened^24^. Also, conflict-affected areas are likely to experience delays or difficulties in accessing care, destruction of health care infrastructure, higher case fatality rates, particular challenges during outbreak control activities, and higher levels of poverty^25–27^. Additionally, the presence of unvaccinated or partially vaccinated persons who are displaced to other locations may also result in ongoing disease transmission and/or new pockets of susceptibility in new communities or in refugee camps and settlements^28^. To ensure all persons are protected from preventable infection, it is critical that displaced persons have access to supplemental immunisation activities or other targeted interventions, like mobile clinics, in the locations in which they settle^29^.

Besides conflict, other factors likely contribute to low coverage in northern Nigeria, including Borno state. These include, but are not limited to, lack of availability of immunisation services at facilities and increasing vaccine hesitancy as a result of misconceptions and low health literacy in the region^30,31^. Along with violence and insecurity, these challenges are substantial yet important to overcome to avoid preventable illness and death. Additional investigations, such as on the safe access and readiness of health facilities, will be critical to examine the entire scope of immunization challenges in Borno.

There are several limitations to this work. First, the production of reliable state-level coverage estimates requires reliable population estimates. If available population estimates do not capture internal migration, i.e., away from conflict areas, then modelled coverage estimates will be subject to bias. A better understanding of subnational patterns of mobility and migration is critical to better understand coverage in conflict areas. Additionally, due to limitations in survey design, the DHS and MICS/NICS surveys were unlikely to capture children displaced and living in temporary settlements. Our estimates are therefore also not representative of these vulnerable populations. Surveys explicitly sampling from these subpopulations are needed in order to assess coverage among these children. We additionally were not able to distinguish between which specific LGAs were unsampled due to security concerns or to those never planned in the survey sampling design. Second, in this analysis, all ACLED conflict types were grouped together to illustrate the overall magnitude of conflict. Different types of conflict may differentially impede both survey sampling and the ability to deliver vaccination services and could be an area for future exploration. Last, mortality rates due to war and terrorism are accounted for at the national level in our models, but no subnational conflict patterns are currently used as predictors of coverage. Further work to define the relationship between vaccine coverage and conflict type and proximity may allow for better estimation of vaccine coverage in conflict areas.

Our geospatial model estimates vaccine coverage in areas unsampled due to conflict but requires the substantial assumption that coverage in conflict areas can be predicted using historical trends, data from surrounding areas, and available covariates. No model of coverage in conflict settings is a substitute, however, for reliable data. To ensure that all children can be reached by vaccination services, all children – including those living in conflict settings – must be represented in estimates of vaccine coverage. Innovative, targeted, and subnationally-representative data collection efforts will be required to overcome conflict-associated challenges of remoteness, security, and political resistance. Examples of these kinds of surveys include but are not limited to the SMART series and other country-specific initiatives^21^. The COVID-19 pandemic may also present specific unique challenges for ongoing household-based data collection, and innovative solutions will be needed to ensure that populations affected by conflict are included.

Understanding vaccination coverage in conflict settings is an equity priority. To reach every child with immunisation services, reliable estimates of vaccination coverage in conflict locations are needed. Innovative model-based coverage estimates, the expansion of new survey methodologies, and reliable population estimates will all be critical tools to ensure that vulnerable children living in conflict-affected areas will be protected against preventable disease and death.

## Methods

We searched for household-based surveys containing information on DTP vaccination status from either vaccination card or parental recall, age, and geographic information via the Global Health Data Exchange (GHDx) from 2000 to 2020^32^. Surveys were included if they contained DTP coverage information and subnational geolocation; surveys were excluded if they did not contain information on children aged 12–59 months, contained no subnational geographic information, contained areal data and were missing key survey design variables (strata, primary sampling units, and design weights), or if coverage estimates were unrealistic (Supplementary Table 3).

In Nigeria, this yielded eight surveys from 109,487 individual children across 5668 specific GPS-located clusters and 843 areally located geographic units. To better capture covariate effects and the potential for vaccine coverage patterns to cross borders, particularly in conflict settings where political boundaries may not be enforced, we modelled coverage in Nigeria as part of a 19-country region representing western sub-Saharan Africa, including an additional 86 surveys from 374,592 individual children.

At the most geographically granular level possible, we calculated DTP1 and DTP3 coverage among 12–59-month-olds, defined as the proportion of children who have received at least one or three doses of DTP, from each source for each birth cohort of children, respectively. Using these survey data and a suite of 26 geospatial and four national covariates (Supplementary Information Tables 2–4), we estimated DTP1 and DTP3 coverage in a Bayesian continuation ratio ordinal regression model-based geostatistical framework that has previously been described in detail^1,2^.

In brief, this model produces spatiotemporal estimates of dose-specific DTP coverage under the assumption that coverage is similar in location-years that have similar covariate patterns and that are close together in space and time. First, we employed a variance inflation factor (VIF) algorithm to reduce multicollinearity amongst the covariates (Supplementary Table 4). Next, to allow for complex and non-linear interactions and to improve predictive accuracy, we used a stacked ensemble modeling approach. Three child models – generalized additive models, lasso regression, and boosted regression trees – were used to model possible surfaces of dose-specific coverage probabilities using the covariates selected by the VIF algorithm as predictors. Then, we modelled the probability of dose-specific vaccination using a hierarchical logistic Bayesian geostatistical model that accounts for variation in coverage due to spatial and temporal correlation as well as covariate patterns. This second-step Bayesian geostatistical model uses a generalized linear regression that models the probability of dose-specific coverage in binomial space with a logit link. The model uses the outcomes from the stacked generalization model as predictors, accounts for observation-specific irreducible error, and includes specific spatial and temporal terms. Additional spatiotemporal residuals were included to account for any remaining variation beyond the predictive capability of modelled covariates, country-specific random effects, and observation-specific irreducible error. A correlated spatiotemporal error term, to account for any residual autocorrelation across space, was modelled via a three-dimensional spatiotemporal Gaussian process with mean zero and covariance equal to the Kronecker product of temporal and spatial covariance kernels. Temporal covariance was modelled via an autoregressive order 1 function and spatial covariance was assumed to be an isotropic, stationary Matérn function. Models were fit in R version 3.5.0 and with the R-INLA package^33,34^.

This modelling framework produced 1000 Bayesian posterior samples of coverage estimates for each 5 × 5-km^2^ area for both DTP1 and DTP3 coverage in each year between 2000 and 2020. Results were calibrated to national-level estimates from the Global Burden of Diseases, Injuries, and Risk Factors Study^3^. Geospatial estimates were aggregated as population-weighted means to first- and second-level administrative units (states and local government area (LGAs), respectively), using population estimates from the WorldPop project and a modified version of the Database of Global Administrative Areas (GADM) shapefile^35,36^. The modelling framework used in this analysis has been validated previously using spatially stratified five-fold out-of-sample cross-validation and has yielded high predictive validity, with 95% coverage of predictive intervals of 92.5% for DTP3 coverage and 98.9% for first-dose measles-containing vaccine (MCV1) coverage and mean error of 0.1% for DTP3 coverage and -0.1% for MCV1 coverage^1,2^.

After modelling, we compared state-level aggregated coverage estimates in Borno state to those reported by recent household-based surveys. We overlaid spatially located conflict reports from the Armed Conflict Location & Event Data Project (ACLED) on our estimates of vaccine coverage and sampling locations from the 2016–17 Multiple Indicators Cluster Survey with National Immunization Coverage Survey supplement (MICS/NICS) and the 2018 Demographic and Health Survey (DHS) to establish if surveys were able to sample from locations likely impacted by conflict^4,9,10^. Additionally, we compared differences in coverage estimates from states affected by conflict and those that were not (Supplementary Figs. 2–5).

We also used subnational gridded population estimates from GRID3 and WorldPop to assess the proportion of the population in Borno state represented among sampled LGAs and the robustness of modelled state-level coverage estimates to variation in LGA-level population estimates^35,37^. This work follows the Guidelines for Accurate and Transparent Health Estimates Reporting (GATHER) recommendations^38^. Additional details on data (http://ghdx.healthdata.org/record/ihme-data/nigeria-dtp-vaccine-coverage-estimates-2000-2018) and code (http://github.com/ihmeuw/vaccines_borno_case_study) have been archived.

## Supplementary Information

Supplementary Information.

## Data Availability

A detailed list of publicly available data sources can be found in Supplementary Tables 2, 3; data may be obtained upon request from the data provider. Administrative boundaries have been modified from the Database for Global Administrative Areas (GADM) dataset^36^. Gridded-population estimates produced by the WorldPop^35^ and GRID3^37^ projects are publicly available online.

## Code availability

Because this study is GATHER compliant^38^ (Supplementary Table 1), all computer code is available from GitHub (http://github.com/ihmeuw/vaccines_borno_case_study). All figures and maps were produced by the authors using RStudio (R version 3.6.1).

## References

[1] Mosser JF (2019). Mapping diphtheria-pertussis-tetanus vaccine coverage in Africa, 2000–2016: A spatial and temporal modelling study. Lancet.

[2] Sbarra AN (2021). Mapping routine measles vaccination in low- and middle-income countries. Nature.

[3] Vos T (2020). Global burden of 369 diseases and injuries in 204 countries and territories, 1990–2019: A systematic analysis for the Global Burden of Disease Study 2019. Lancet.

[4] ACLED. *ACLED* https://acleddata.com/.

[5] Grundy J, Biggs B-A (2018). The impact of conflict on immunisation coverage in 16 countries. Int. J. Health Policy Manag..

[6] Drapcho, B. & Mock, N. *DHS as a Survey Vehicle in Conflict Settings Draft Working Paper* (2000).

[7] Axinn WG, Ghimire D, Williams NE (2012). Collecting survey data during armed conflict. J. Off. Stat..

[8] ERG Discussion Paper 6 Conflict 24 March 2019.pdf. https://drive.google.com/file/u/1/d/1R7BecCx_JGxIAZQVcQDtzhAo_La2ASJ5/view?usp=embed_facebook.

[9] Unicef MICS. Multiple indicator cluster survey 2016–17 (MICS). https://www.unicef.org/nigeria/reports/multiple-indicator-cluster-survey-2016-17-mics.

[10] National Population Commission, The DHS Program. Nigeria Demographic and Health Survey 2018 - Final Report. https://dhsprogram.com/publications/publication-fr359-dhs-final-reports.cfm (2019).

[11] Local Burden of Disease Child Growth Failure Collaborators (2020). Mapping child growth failure across low- and middle-income countries. Nature.

[12] Burstein R (2019). Mapping 123 million neonatal, infant and child deaths between 2000 and 2017. Nature.

[13] Dwyer-Lindgren L (2019). Mapping HIV prevalence in sub-Saharan Africa between 2000 and 2017. Nature.

[14] Gavi, The Vaccine Alliance. 2016–2020 Strategy Indicator Definitions. https://www.gavi.org/sites/default/files/document/gavi-2016-2020-strategy-indicator-definitionspdf.pdf.

[15] Cutts FT, Claquin P, Danovaro-Holliday MC, Rhoda DA (2016). Monitoring vaccination coverage: Defining the role of surveys. Vaccine.

[16] Jacobsen K, Landau LB (2003). The dual imperative in refugee research: Some methodological and ethical considerations in social science research on forced migration. Disasters.

[17] Cohen N, Arieli T (2011). Field research in conflict environments: Methodological challenges and snowball sampling. J. Peace Res..

[18] International Organization for Migration. Global DTM Website. https://displacement.iom.int/.

[19] WHO and partners support measles vaccination in Borno State, Nigeria. https://www.who.int/news-room/feature-stories/detail/who-and-partners-support-measles-vaccination-in-borno-state-nigeria.

[20] Bawa S (2019). Using the polio programme to deliver primary health care in Nigeria: Implementation research. Bull. World Health Organ..

[21] About SMART. *SMART Methodology*. https://smartmethodology.org/about-smart/.

[22] Babakura B (2021). The challenges of insecurity on implementing vaccination campaign and its effect on measles elimination and control efforts: A case study of 2017/18 measles campaign in Borno state Nigeria. Vaccine.

[23] Besa NC (2014). Diphtheria outbreak with high mortality in northeastern Nigeria. Epidemiol. Infect..

[24] Statement of the 14th IHR Emergency Committee regarding the international spread of poliovirus. https://www.who.int/news/item/03-08-2017-statement-of-the-14th-ihr-emergency-committee-regarding-the-international-spread-of-poliovirus.

[25] Raad II, Chaftari A-M, Dib RW, Graviss EA, Hachem R (2018). Emerging outbreaks associated with conflict and failing healthcare systems in the Middle East. Infect. Control Hosp. Epidemiol..

[26] O’Hare BAM, Southall DP (2007). First do no harm: The impact of recent armed conflict on maternal and child health in Sub-Saharan Africa. J. R. Soc. Med..

[27] Gates S, Hegre H, Nygård HM, Strand H (2012). Development consequences of armed conflict. World Dev..

[28] Connolly MA, Gayer M, Ryan MJ, Salama P, Spiegel P, Heymann DL (2004). Communicable diseases in complex emergencies: Impact and challenges. Lancet.

[29] Lam E, McCarthy A, Brennan M (2015). Vaccine-preventable diseases in humanitarian emergencies among refugee and internally-displaced populations. Hum. Vaccines Immunother..

[30] Akwataghibe NN (2019). Exploring factors influencing immunization utilization in Nigeria—A mixed methods study. Front. Public Health.

[31] Galadima A, Mohd Zulkefli N, Md S, Ahmad N (2020). Factors influencing childhood immunization uptake in Africa: A systematic review. BMC Public Health.

[32] Global Health Data Exchange | GHDx. http://ghdx.healthdata.org/.

[33] The Comprehensive R Archive Network. https://cran.r-project.org/.

[34] The R-INLA project. http://www.r-inla.org/.

[35] Tatem AJ (2017). WorldPop, open data for spatial demography. Sci. Data.

[36] GADM. https://gadm.org/.

[37] GRID3. *GRID3* https://grid3.org/.

[38] Stevens GA (2016). Guidelines for accurate and transparent health estimates reporting: The GATHER statement. Lancet.

