# Supplementary material for: Estimating vaccine coverage in conflict settings using geospatial methods: a case study in Borno state, Nigeria
Source: Sci Rep. 2023 Jul 8;13:11085. doi: 10.1038/s41598-023-37947-8 (PMC10329660; doi:10.1038/s41598-023-37947-8)
Supplement: Supplementary file 1 — Supplementary Information. [file 41598_2023_37947_MOESM1_ESM.pdf]

Estimating vaccine coverage in conflict settings using geospatial methods: a case study in Borno state, Nigeria

1.0 Supplementary Results 2

2.0 Supplementary Figures 3

Supplementary Figure 1. *Locations and types of conflict in Nigeria and Borno state.* 3

Supplementary Figure 2a. *Relative difference in coverage between MBG estimates and reported survey coverage from 2016-17 MICS/NICS, DTP1.* 4

Supplementary Figure 2b. *Relative difference in coverage between MBG estimates and reported survey coverage from 2016-17 MICS/NICS, DTP3.* 5

Supplementary Figure 3a. *Relative difference in coverage between MBG estimates and reported survey coverage from 2018 DHS, DTP1.* 6

Supplementary Figure 3b. *Relative difference in coverage between MBG estimates and reported survey coverage from 2018 DHS, DTP3.* 7

Supplementary Figure 4a. *Ranked relative difference between reported survey coverage and modelled estimates from 2016-17 MICS/NICS.* 8

Supplementary Figure 4b. *Ranked relative difference between reported survey coverage and modelled estimates from 2018 DHS.* 9

Supplementary Figure 5. *Mapped difference in coverage estimates between survey and modelled results.* 10

3.0 Supplementary Tables 11

Supplementary Table 1: GATHER compliance checklist 11

Supplementary Table 2: Input sources included in final analysis in Nigeria 13

Supplementary Table 3: Survey sources excluded from analysis 23

Supplementary Table 4: Final covariate set used for modelling 30

Supplementary References 31

1.0 Supplementary Results

We examined all classifications of conflict types in Nigeria from the ACLED database[1]; these types include battles, explosions/remote violence, protests, riots, strategic developments, and violence against civilians. Frequency and classification of conflicts vary subnationally (Supplementary Fig 1). For the remainder of analysis, we aggregated conflicts across type and considered any classification of conflict as a conflict event.

We additionally compared modelled estimates to the availability of surveys to sample in various subnational units. Additional figures can be found in Supplementary Figs. 2-5. For this analysis, we considered states affected by conflict to be Borno, Yobe, Taraba, and Adamawa. Borno was the state whose sampling was most affected across the two surveys, followed by Yobe particularly in the 2016-17 MICS/NICS survey (Table 1 in main text). We compared the relative differences in coverage between surveys and modelled estimates across conflict-affected and unaffected states and also how these differences varied by underlying coverage values (Supplementary Figs. 3-5).

This analysis showed that there are overall differences between the modelled coverage estimates and the survey results. Generally, these differences can be attributable to the modelling approach, which beyond the specific surveys used for comparison, additionally uses surveys from multiple years and additionally incorporate national-level administrative data in the estimation process. For example, in the comparison of the MICS/NICS survey results to the modelled estimates for both DTP1 and DTP3, we found modelled estimates to be higher than the survey results in most states (Supplementary Figs. 2 and 4a). Borno, however, was a marked outlier, with modelled coverage estimates being much lower.

This same pattern was less striking while comparing modelled estimates to DHS survey results (Supplementary Figs. 3 and 4b), but still Borno had some of the larger negative differences when compared to the rest of the states. We also assessed differences in modelled coverage estimates and survey results across three additional conflict affected states (Yobe, Taraba and Adamawa). Patterns observed were less clear. However, as survey sampling was largely able to proceed as planned in these states despite conflict, this may be expected. These results overall suggest that variations observed in differences between survey- and model-based coverage estimates could also be related to underlying differences in methodologies, in addition to known limitations related to sampling representativeness.

2.0 Supplementary Figures

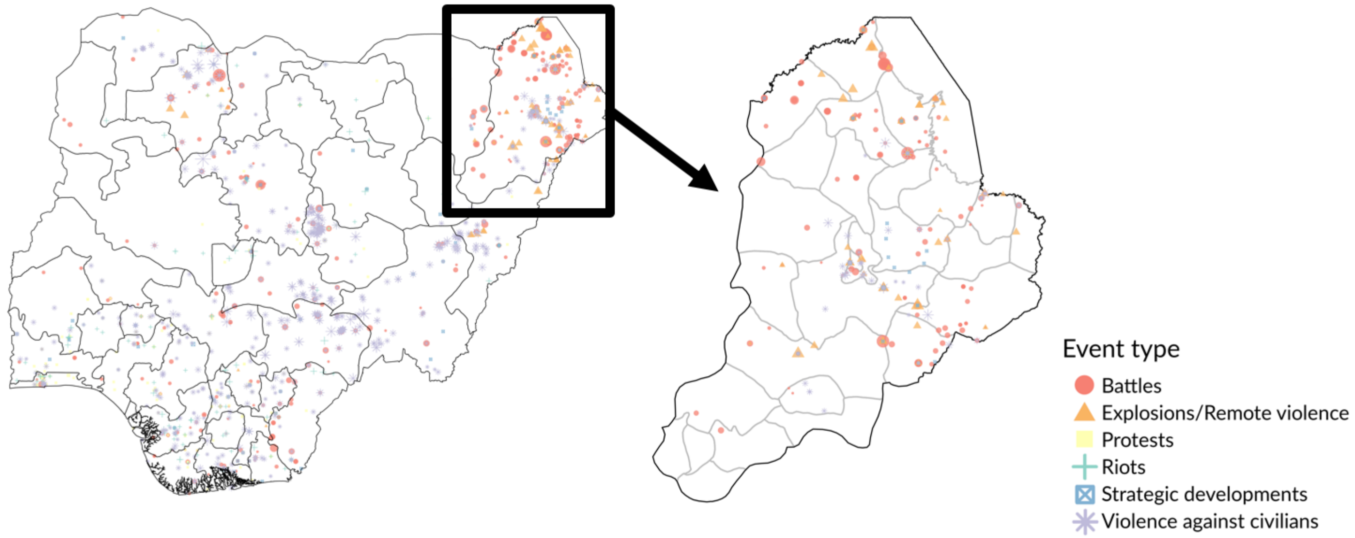

Supplementary Figure 1. *Locations and types of conflict in Nigeria and Borno state.*
Per the Armed Conflict Location & Event Data Project (ACLED) database, in 2018, the locations and types of conflict events in Nigeria and Borno state are shown. Pink circles depict battle events, orange triangles depict explosions or remote violence, yellow squares depict protests, green crosses depict riots, blue-crossed squares depict strategic developments, and purple stars depict violence against civilians. All event types are sized by number of fatalities per event. Maps were produced in R version 3.5.0 (https://cran.r-project.org/).

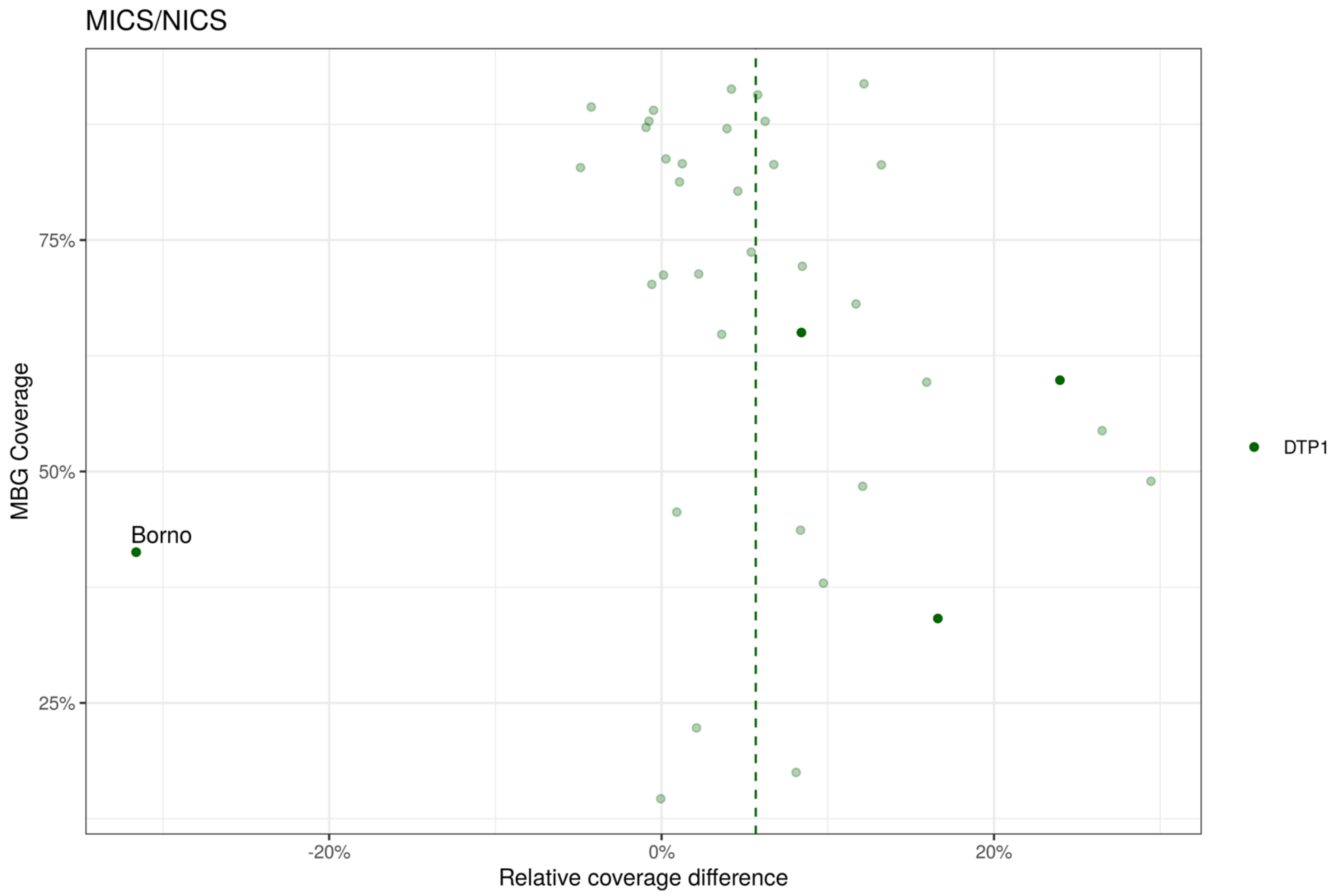

Supplementary Figure 2a. *Relative difference in coverage between MBG estimates and reported survey coverage from 2016-17 MICS/NICS, DTP1.*
Modelled DTP1 (green) coverage versus relative difference in coverage between MBG and survey results is shown. States affected by conflict are emphasised in darker colours. The mean relative difference is shown in the dashed vertical line.

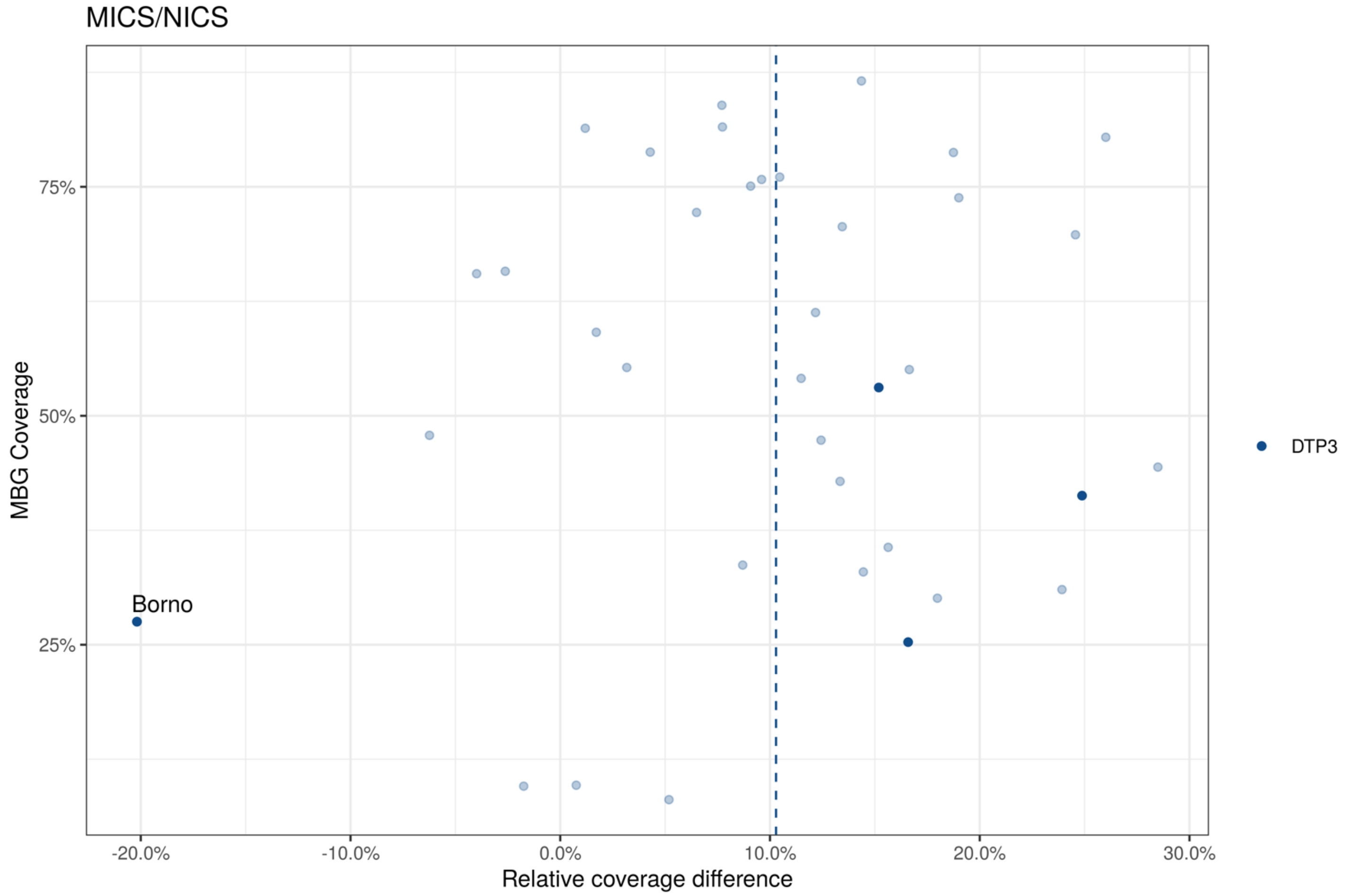

Supplementary Figure 2b. *Relative difference in coverage between MBG estimates and reported survey coverage from 2016-17 MICS/NICS, DTP3.*
Modelled DTP3 (blue) coverage versus relative difference in coverage between MBG and survey results is shown. States affected by conflict are emphasised in darker colours. The mean relative difference is shown in the dashed vertical line.

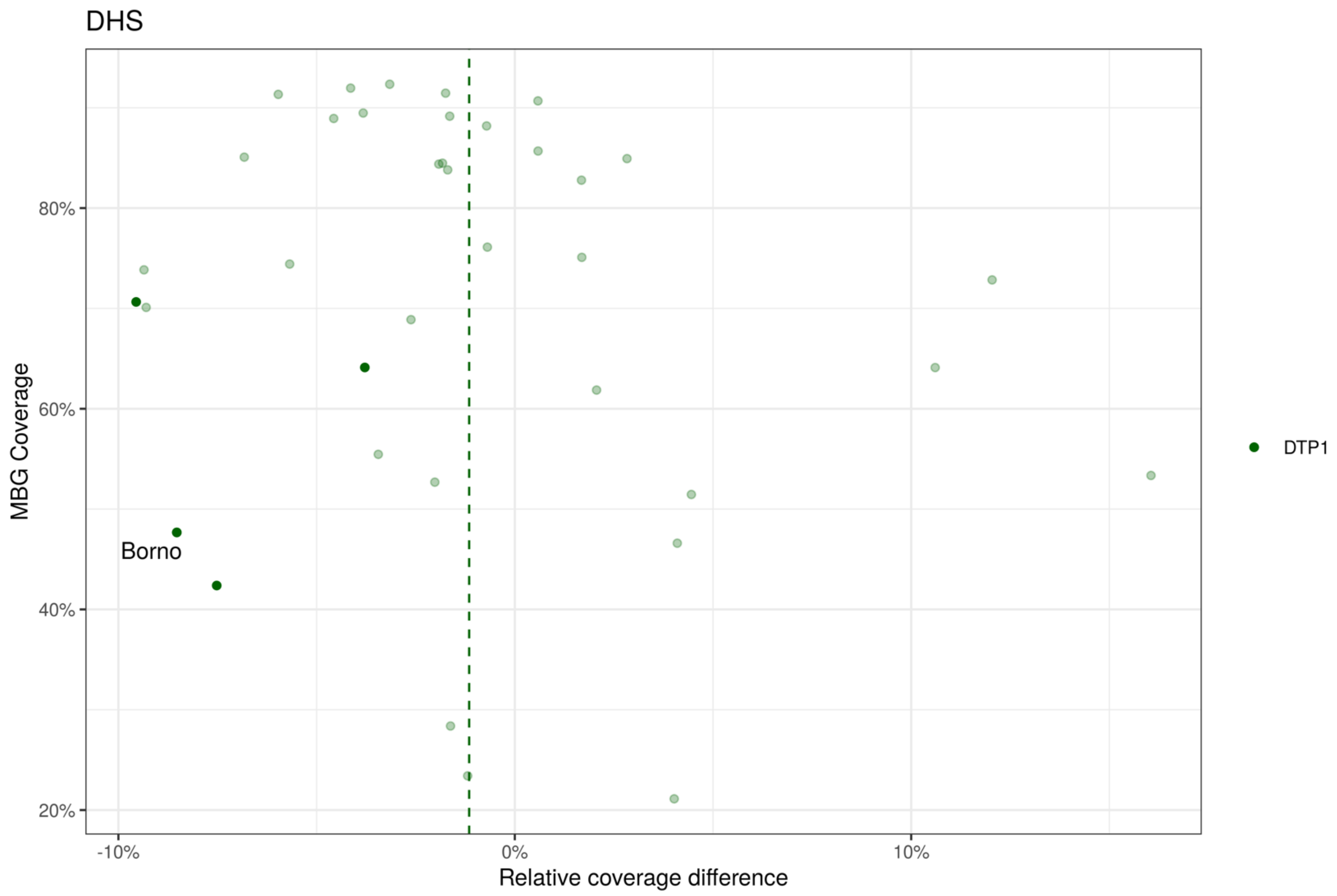

Supplementary Figure 3a. *Relative difference in coverage between MBG estimates and reported survey coverage from 2018 DHS, DTP1.*
Modelled DTP1 (green) coverage versus relative difference in coverage between MBG and survey results is shown. States affected by conflict are emphasised in darker colours. The mean relative difference is shown in the dashed vertical line.

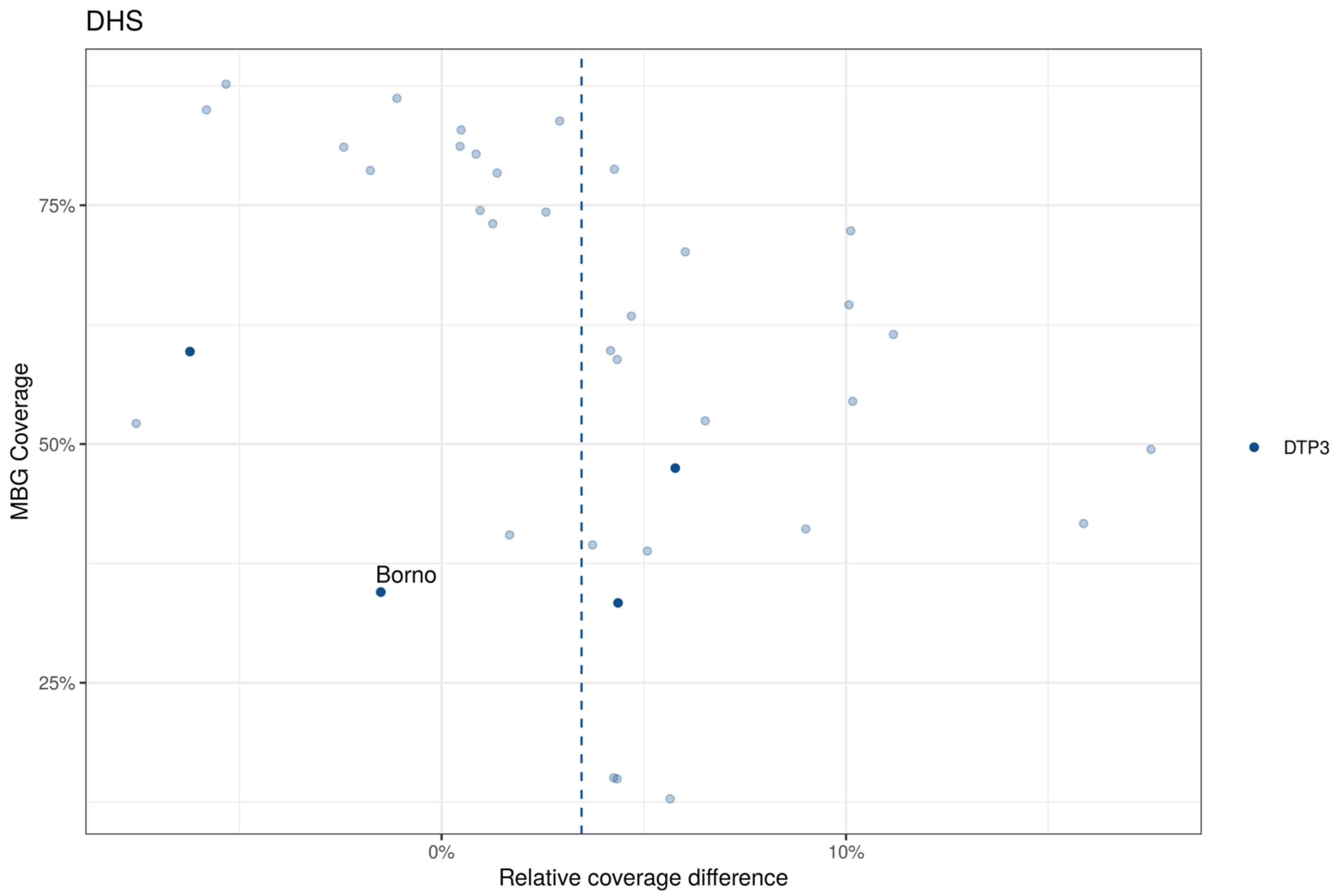

Supplementary Figure 3b. *Relative difference in coverage between MBG estimates and reported survey coverage from 2018 DHS, DTP3.*
Modelled DTP3 (blue) coverage versus relative difference in coverage between MBG and survey results is shown. States affected by conflict are emphasised in darker colours. The mean relative difference is shown in the dashed vertical line.

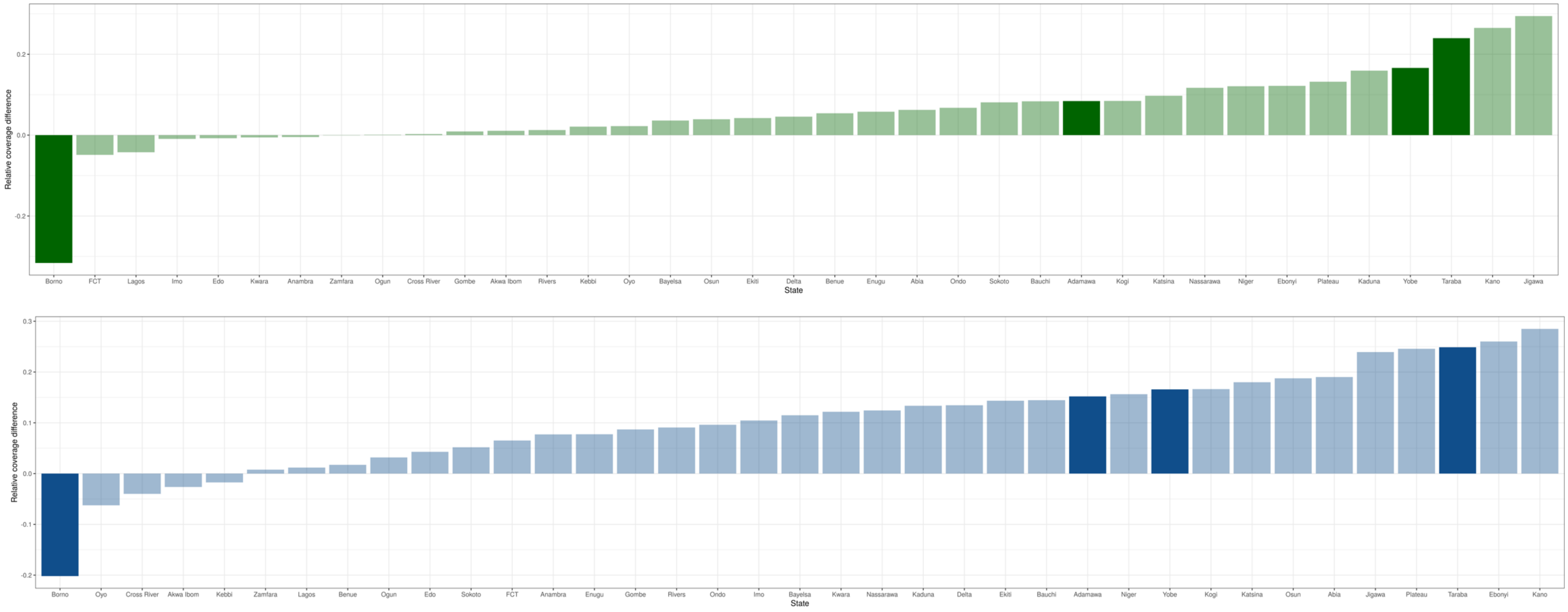

Supplementary Figure 4a. *Ranked relative difference between reported survey coverage and modelled estimates from 2016-17 MICS/NICS.*
Relative difference in coverage between survey-reported results and modelled estimates for DTP1 (green, top) and DTP3 (blue, bottom) are shown. States affected by conflict are emphasised in darker colours.

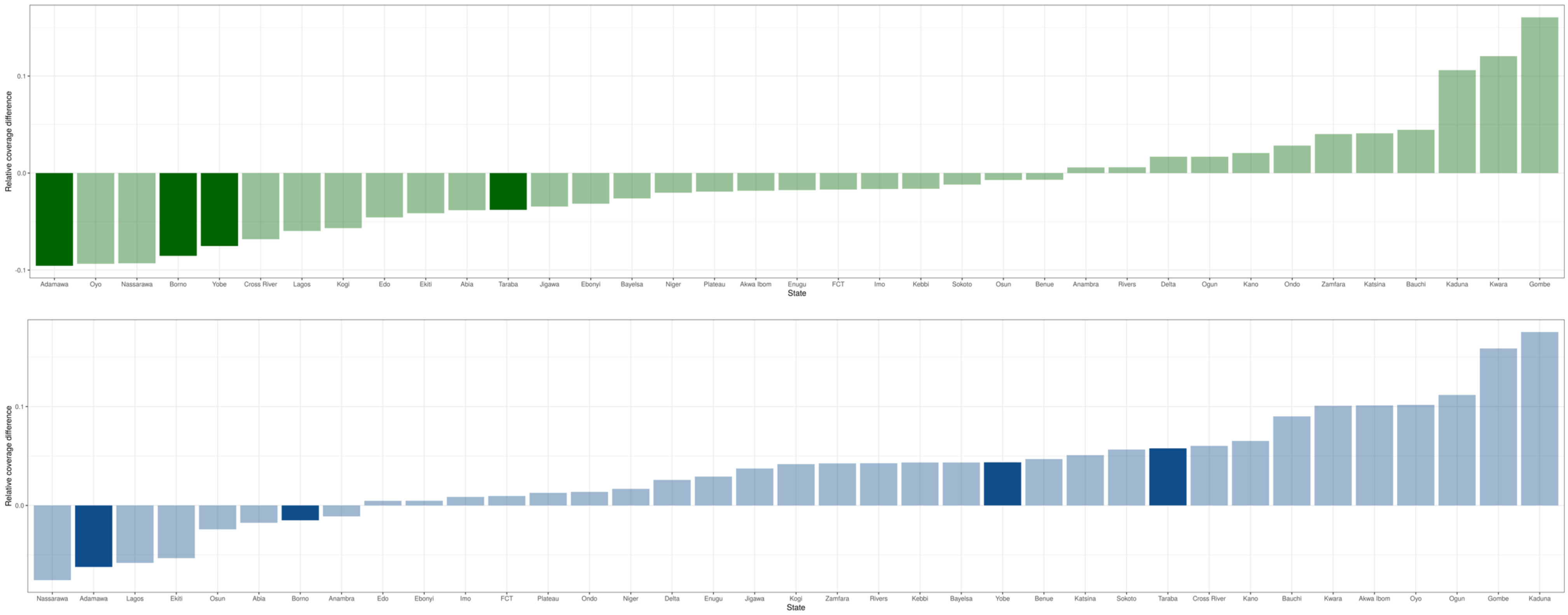

Supplementary Figure 4b. *Ranked relative difference between reported survey coverage and modelled estimates from 2018 DHS.* Relative difference in coverage between survey-reported results and modelled estimates for DTP1 (green, top) and DTP3 (blue, bottom) are shown. States affected by conflict are emphasised in darker colours.

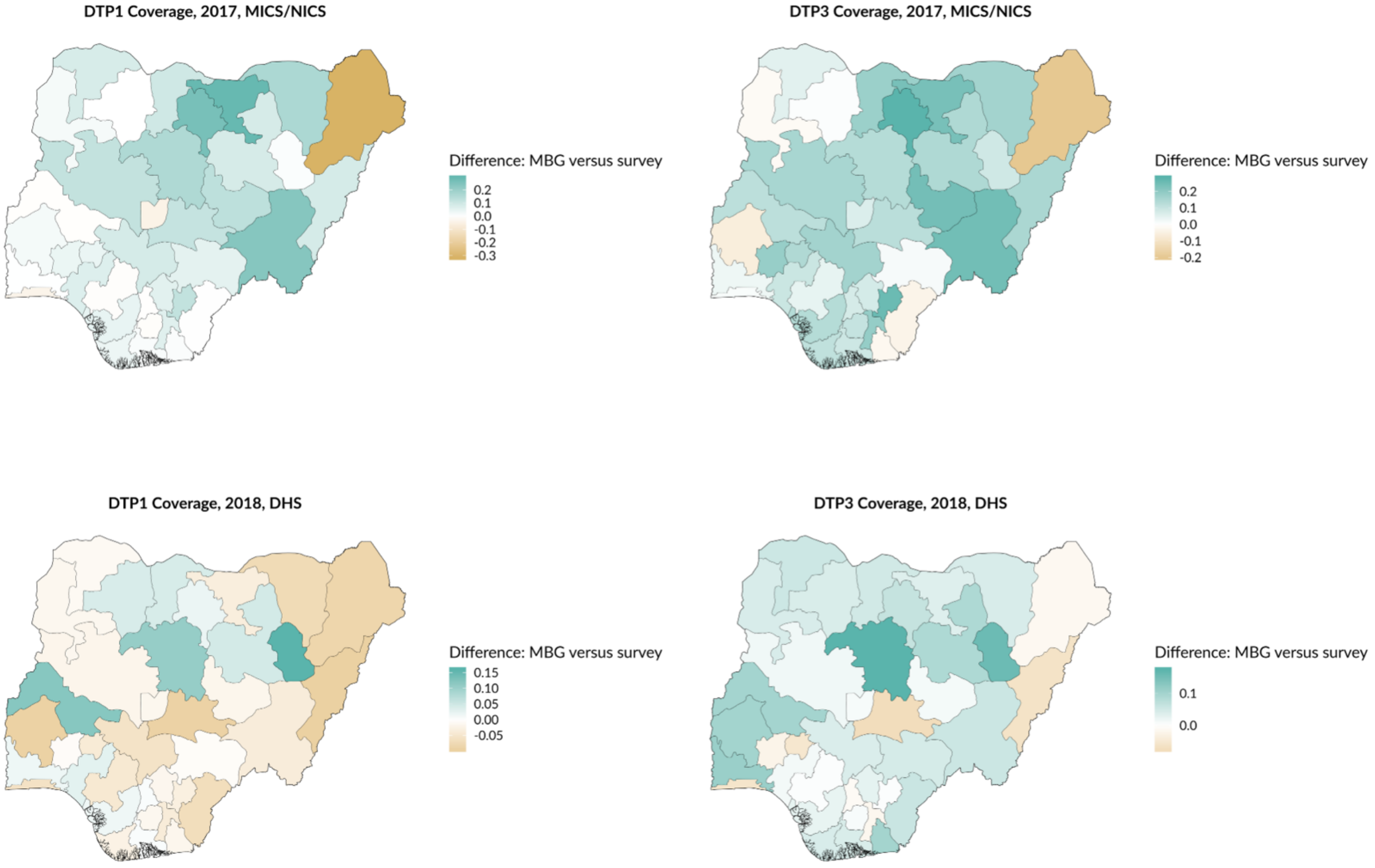

Supplementary Figure 5. *Mapped difference in coverage estimates between survey and modelled results.*
Difference in coverage between survey reported results and modelled estimates for DTP1 and DTP3 is shown for both 2016-17 MICS/NICS and 2018 DHS. Maps were produced in R version 3.5.0 (https://cran.r-project.org/).

3.0 Supplementary Tables

Supplementary Table 1: GATHER compliance checklist

Item number	Checklist item	Reported on page number(s):
**Objectives and funding**		
1	Define the indicator(s), populations (including age, sex, and geographic entities), and time period(s) for which estimates were made.	Methods
2	List the funding sources for the work.	Acknowledgments
**Data inputs**		
For all data inputs from multiple sources that are synthesised as part of the study:		
3	Describe how the data were identified and how the data were accessed.	Methods
4	Specify the inclusion and exclusion criteria. Identify all ad hoc exclusions.	Methods
5	Provide information on all included data sources and their main characteristics. For each data source used, report reference information or contact name/institution, population represented, data collection method, year(s) of data collection, sex and age range, diagnostic criteria or measurement method, and sample size, as relevant.	Supplementary Tables 2-3
6	Identify and describe any categories of input data that have potentially important biases (e.g., based on characteristics listed in item 5).	Supplementary Table 2-4
For data inputs that contribute to the analysis but were not synthesised as part of the study:		
7	Describe and give sources for any other data inputs.	Methods
For all data inputs:		
8	Provide all data inputs in a file format from which data can be efficiently extracted (e.g., a spreadsheet rather than a PDF), including all relevant meta-data listed in item 5. For any data inputs that cannot be shared because of ethical or legal reasons, such as third-party ownership, provide a contact name or the name of the institution that retains the right to the data.	Supplementary Table 2-3; http://ghdx.healthdata.org/lbd-publication-data-input-sources
**Data analysis**		

9	Provide a conceptual overview of the data analysis method. A diagram might be helpful.	Methods
10	Provide a detailed description of all steps of the analysis, including mathematical formulae. This description should cover, as relevant, data cleaning, data pre-processing, data adjustments and weighting of data sources, and mathematical or statistical model(s).	Methods
11	Describe how candidate models were evaluated and how the final model(s) were selected.	Methods
12	Provide the results of an evaluation of model performance, if done, as well as the results of any relevant sensitivity analysis.	Methods
13	Describe methods for calculating uncertainty of the estimates. State which sources of uncertainty were, and were not, accounted for in the uncertainty analysis.	Methods
14	State how analytic or statistical source used to generate estimates can be accessed.	Data availability; Code availability
**Results and discussion**		
15	Provide published estimates in a file format from which data can be efficiently extracted.	Data availability
16	Report a quantitative measure of uncertainty of the estimates (e.g., uncertainty intervals).	Results
17	Interpret results in light of existing evidence. If updating a previous set of estimates, describe the reasons for changes in estimates.	Discussion
18	Discuss limitations that affect interpretation of the estimates.	Discussion

Supplementary Table 2: Input sources included in final analysis in Nigeria

List of sources with GHDx identification number included in the final analysis is provided for surveys in Nigeria. These sources can also be explored following this link to the GHDx input data sources tool: http://ghdx.healthdata.org/lbd-publication-data-input-sources.

GHDx id	Country	Series	Year(s)	Geoprecision	Citation	Link
9516	Nigeria	Nigeria Multiple Indicator Cluster Survey 2007	2007	Admin1	United Nations Children's Fund (UNICEF), National Bureau of Statistics (Nigeria). Nigeria Multiple Indicator Cluster Survey 2007. New York, United States: United Nations Children's Fund (UNICEF).	GHDx
20567	Nigeria	Nigeria Demographic and Health Survey 2003	2003	Latitude/longitude	Department for International Development (DFiD) (United Kingdom), National Population Commission of Nigeria, ORC Macro, United Nations Children's Fund (UNICEF), United Nations Population Fund (UNFPA). Nigeria Demographic and Health Survey 2003. Fairfax, United States of America: ICF International.	GHDx
21433	Nigeria	Nigeria Demographic and Health Survey 2008	2008	Latitude/longitude	Macro International, Inc, National Population Commission of Nigeria. Nigeria Demographic and Health Survey 2008. Fairfax, United States of America: ICF International, 2009.	GHDx
76703	Nigeria	Nigeria Multiple Indicator Cluster Survey 2011	2011	Admin1	National Bureau of Statistics (Nigeria), United Nations Children's Fund (UNICEF). Nigeria Multiple Indicator Cluster Survey 2011. New York, United States of America: United Nations Children's Fund (UNICEF), 2013.	GHDx
77390	Nigeria	Nigeria Demographic and Health Survey 2013	2013	Latitude/longitude	ICF International, National Population Commission of Nigeria. Nigeria Demographic and Health Survey 2013. Fairfax, United States of America: ICF International.	GHDx
151719	Nigeria	Nigeria Living Standards Survey 2008-2010	2008-2010	Admin2	National Bureau of Statistics (Nigeria). Nigeria Living Standards Survey 2008-2010. Abuja, Nigeria: National Bureau of Statistics (Nigeria).	GHDx
408484	Nigeria	Nigeria Demographic and Health Survey 2018	2018	Latitude/longitude	Federal Ministry of Health (Nigeria), ICF International, National Population Commission (NPC). Nigeria Demographic and Health Survey 2018. Fairfax, United States of America: ICF International, 2020.	GHDx
437724	Nigeria	Nigeria Multiple Indicator Cluster Survey with National Immunization Coverage Survey Supplement 2016-2017 - National Bureau of Statistics	2016-2017	Latitude/longitude	National Agency for the Control of AIDS (Nigeria), National Bureau of Statistics (Nigeria), National Primary Health Care Development Agency (NPHCDA) (Nigeria), United Nations Children's Fund (UNICEF). Nigeria Multiple Indicator Cluster Survey with National Immunization Coverage Survey Supplement 2016-2017 - National Bureau of Statistics. New York, United States of America: United Nations Children's Fund (UNICEF), 2018.	GHDx

Supplementary Table 3: Input sources included in final analysis in western sub-Saharan Africa region

List of remaining survey sources with GHDx identification number included in the final analysis is provided for surveys in the rest of western sub-Saharan Africa. These sources can also be explored following this link to the GHDx input data sources tool: http://ghdx.healthdata.org/lbd-publication-data-input-sources.

GHDx id	Country	Series	Year(s)	Geoprecision	Citation	Link
18950	Benin	Benin Demographic and Health Survey 2001	2001	Latitude/longitude	National Institute of Statistics and Economic Analysis (INSAE) (Benin), ORC Macro. Benin Demographic and Health Survey 2001. Fairfax, United States of America: ICF International.	GHDx
18959	Benin	Benin Demographic and Health Survey 2006	2006	Admin3	Macro International, Inc, National Institute of Statistics and Economic Analysis (INSAE) (Benin), National Program Against AIDS (PNLS) (Benin). Benin Demographic and Health Survey 2006. Fairfax, United States of America: ICF International.	GHDx
206075	Benin	Benin Multiple Indicator Cluster Survey 2014	2014	Admin1	National Institute of Statistics and Economic Analysis (INSAE) (Benin), United Nations Children's Fund (UNICEF). Benin Multiple Indicator Cluster Survey 2014. New York, United States of America: United Nations Children's Fund (UNICEF), 2017.	GHDx
218565	Benin	Benin Demographic and Health Survey 2017-2018	2017-2018	Latitude/longitude	Hubert Koutoukou Maga National University Hospital Center (CNHU-HKM) (Benin), ICF International, National Institute of Statistics and Economic Analysis (INSAE) (Benin), National Malaria Control Program, Ministry of Health (Benin), Permanent Secretariat of the Food Council and Nutrition (SP-CAN) (Benin). Benin Demographic and Health Survey 2017-2018. Fairfax, United States of America: ICF International, 2018.	GHDx
1927	Burkina Faso	Burkina Faso Multiple Indicator Cluster Survey 2006	2006	Admin1	National Institute of Statistics and Demography (Burkina Faso), United Nations Children's Fund (UNICEF). Burkina Faso Multiple Indicator Cluster Survey 2006. New York, United States of America: United Nations Children's Fund (UNICEF).	GHDx
19088	Burkina Faso	Burkina Faso Demographic and Health Survey 2003	2003	Latitude/longitude	Macro International, Inc, National Institute of Statistics and Demography (Burkina Faso). Burkina Faso Demographic and Health Survey 2003. Fairfax, United States of America: ICF International.	GHDx
19133	Burkina Faso	Burkina Faso Demographic and Health Survey 2010-2011	2010-2011	Latitude/longitude	ICF Macro, Ministry of Health (Burkina Faso), National Institute of Statistics and Demography (Burkina Faso). Burkina Faso Demographic and Health Survey 2010-2011. Fairfax, United States of America: ICF International.	GHDx
26642	Burkina Faso	Burkina Faso Global Fund Household Health Coverage Survey 2008	2008	Admin3	Global Fund to Fight Aids Tuberculosis and Malaria (GFATM). Burkina Faso Global Fund Household Health Coverage Survey 2008.	GHDx

236156	Burkina Faso	Burkina Faso Continuous Multisectoral Survey 2014	2014	Admin3	National Institute of Statistics and Demography (Burkina Faso), World Bank. Burkina Faso Continuous Multisectoral Survey 2014. Washington DC, United States of America: World Bank.	GHDx
2053	Cameroon	Cameroon Multiple Indicator Cluster Survey 2000	2000	Admin3	Directorate of Statistics and National Accounts, Ministry of Economics and Finance (Cameroon), United Nations Children's Fund (UNICEF). Cameroon Multiple Indicator Cluster Survey 2000. New York, United States of America: United Nations Children's Fund (UNICEF).	GHDx
2063	Cameroon	Cameroon Multiple Indicator Cluster Survey 2006	2006	Admin3	United Nations Children's Fund (UNICEF), National Institute of Statistics (Cameroon). Cameroon Multiple Indicator Cluster Survey 2006. New York, United States: United Nations Children's Fund (UNICEF).	GHDx
19211	Cameroon	Cameroon Demographic and Health Survey 2004	2004	Latitude/longitude	Macro International, Inc, National Institute of Statistics (Cameroon). Cameroon Demographic and Health Survey 2004. Fairfax, United States of America: ICF International.	GHDx
19274	Cameroon	Cameroon Demographic and Health Survey 2011	2011	Latitude/longitude	ICF International, Ministry of Economy, Planning and Regional Development (Cameroon), Ministry of Public Health (Cameroon), National Institute of Statistics (Cameroon), Pasteur Center of Cameroon. Cameroon Demographic and Health Survey 2011. Fairfax, United States of America: ICF International.	GHDx
244455	Cameroon	Cameroon Multiple Indicator Cluster Survey 2014	2014	Admin1	Ministry of Public Health (Cameroon), National Institute of Statistics (Cameroon), United Nations Children's Fund (UNICEF). Cameroon Multiple Indicator Cluster Survey 2014. New York, United States of America: United Nations Children's Fund (UNICEF), 2017.	GHDx
413167	Cameroon	Cameroon Demographic and Health Survey 2018-2019	2018-2019	Latitude/longitude	ICF International, Ministry of Public Health (Cameroon), National Institute of Statistics (Cameroon). Cameroon Demographic and Health Survey 2018-2019. Fairfax, United States of America: ICF International.	GHDx
21442	Cape Verde	Cape Verde Demographic and Health Survey 2005	2005	Admin1	Macro International, Inc, Ministry of Health (Cape Verde), National Institute of Statistics (Cape Verde). Cape Verde Demographic and Health Survey 2005.	GHDx
2244	Chad	Chad Multiple Indicator Cluster Survey 2000	2000	Admin1	United Nations Children's Fund (UNICEF), Census Bureau (Chad), National Institute of Statistical, Economic and Demographic Studies (Chad). Chad Multiple Indicator Cluster Survey 2000. New York, United States: United Nations Children's Fund (UNICEF).	GHDx
19315	Chad	Chad Demographic and Health Survey 2004	2004	Admin1	Macro International, Inc, National Institute for Statistics, Economic and Demographic Studies (INSEED) (Chad). Chad Demographic and Health Survey 2004. Fairfax, United States of America: ICF International.	GHDx
76701	Chad	Chad Multiple Indicator Cluster Survey 2010	2010	Admin1	Ministry of Planning, Economy, and International Cooperation (Chad), National Institute for Statistics, Economic and Demographic Studies (INSEED) (Chad), United Nations Children's Fund (UNICEF). Chad	GHDx

					Multiple Indicator Cluster Survey 2010. New York, United States of America: United Nations Children's Fund (UNICEF), 2014.	
157025	Chad	Chad Demographic and Health Survey 2014-2015	2014-2015	Latitude/longitude	ICF International, National Institute for Statistics, Economic and Demographic Studies (INSEED) (Chad). Chad Demographic and Health Survey 2014-2015. Fairfax, United States of America: ICF International, 2016.	GHDx
18533	Côte d'Ivoire	Côte d'Ivoire Demographic and Health Survey 2011-2012	2011-2012	Latitude/longitude	ICF International, Ministry of the Fight Against AIDS (Côte d'Ivoire), National Institute of Statistics (Côte d'Ivoire). Côte d'Ivoire Demographic and Health Survey 2011-2012. Fairfax, United States of America: ICF International.	GHDx
26433	Côte d'Ivoire	Côte d'Ivoire Multiple Indicator Cluster Survey 2006	2006	Admin1	United Nations Children's Fund (UNICEF), National Institute of Statistics (Côte d'Ivoire). Côte d'Ivoire Multiple Indicator Cluster Survey 2006. New York, United States: United Nations Children's Fund (UNICEF).	GHDx
26444	Côte d'Ivoire	Côte d'Ivoire Multiple Indicator Cluster Survey 2000	2000	Admin1	National School for Statistics and Economics Applied (ENSEA), United Nations Children's Fund (UNICEF), United Nations Educational, Scientific and Cultural Organization (UNESCO). Côte d'Ivoire Multiple Indicator Cluster Survey 2000. New York, United States of America: United Nations Children's Fund (UNICEF).	GHDx
3922	Gambia	Gambia Multiple Indicator Cluster Survey 2000	2000	Precise place names	Central Statistics Department (Gambia), United Nations Children's Fund (UNICEF). Gambia Multiple Indicator Cluster Survey 2000. New York, United States: United Nations Children's Fund (UNICEF).	GHDx
3935	Gambia	Gambia Multiple Indicator Cluster Survey 2005-2006	2005-2006	Precise place names	Gambia Bureau of Statistics (GBOS), United Nations Children's Fund (UNICEF). Gambia Multiple Indicator Cluster Survey 2005-2006. New York, United States of America: United Nations Children's Fund (UNICEF).	GHDx
77384	Gambia	Gambia Demographic and Health Survey 2013	2013	Precise place names	Gambia Bureau of Statistics (GBOS), ICF International, Ministry of Health and Social Welfare (The Gambia). Gambia Demographic and Health Survey 2013. Fairfax, United States of America: ICF International, 2015.	GHDx
91506	Gambia	Gambia Multiple Indicator Cluster Survey 2010	2010	Precise place names	Gambia Bureau of Statistics (GBOS), United Nations Children's Fund (UNICEF). Gambia Multiple Indicator Cluster Survey 2010. New York, United States of America: United Nations Children's Fund (UNICEF), 2018.	GHDx
424884	Gambia	Gambia Multiple Indicator Cluster Survey 2018	2018	Precise place names	Gambia Bureau of Statistics (GBOS), United Nations Children's Fund (UNICEF). Gambia Multiple Indicator Cluster Survey 2018. New York, United States of America: United Nations Children's Fund (UNICEF), 2019.	GHDx
459854	Gambia	Gambia Demographic and Health Survey 2019-2020	2019-2020	Precise place names	Gambia Bureau of Statistics (GBOS), ICF International, Ministry of Health and Social Welfare (The Gambia). Gambia Demographic and Health Survey 2019-2020. Fairfax, United States of America: ICF International, 2021.	GHDx
4694	Ghana	Ghana Multiple Indicator Cluster Survey 2006	2006	Admin1	Ministry of Health (MOH) (Ghana), Ghana Statistical Service and United Nations Children's Fund (UNICEF). Ghana Multiple Indicator Cluster Survey 2006. New York, United States: United Nations Children's Fund (UNICEF).	GHDx

19627	Ghana	Ghana Demographic and Health Survey 2003	2003	Latitude/longitude	Ghana Statistical Service, Macro International, Inc. Ghana Demographic and Health Survey 2003. Fairfax, United States of America: ICF International.	GHDx
21188	Ghana	Ghana Demographic and Health Survey 2008	2008	Latitude/longitude	Ghana Statistical Service, Macro International, Inc, Ministry of Health (Ghana). Ghana Demographic and Health Survey 2008. Fairfax, United States of America: ICF International.	GHDx
63993	Ghana	Ghana Multiple Indicator Cluster Survey 2011	2011	Latitude/longitude	Centers for Disease Control and Prevention (CDC), Ghana Statistical Service, Government of Japan, ICF Macro, Ministry of Health (Ghana), Navrongo Health Research Centre, United Nations Children's Fund (UNICEF), United Nations Population Fund (UNFPA), United States Agency for International Development (USAID). Ghana Multiple Indicator Cluster Survey 2011. New York, United States of America: United Nations Children's Fund (UNICEF), 2013.	GHDx
157027	Ghana	Ghana Demographic and Health Survey 2014	2014	Latitude/longitude	Ghana Health Service, Ghana Statistical Service, ICF International. Ghana Demographic and Health Survey 2014. Fairfax, United States of America: ICF International, 2016.	GHDx
437993	Ghana	Ghana Multiple Indicator Cluster Survey 2017-2018	2017-2018	Admin1	Centers for Disease Control and Prevention (CDC), Ghana Statistical Service, Government of Japan, ICF Macro, Ministry of Health (Ghana), Navrongo Health Research Centre, United Nations Children's Fund (UNICEF), United Nations Population Fund (UNFPA), United States Agency for International Development (USAID). Ghana Multiple Indicator Cluster Survey 2017-2018. New York, United States of America: United Nations Children's Fund (UNICEF), 2013.	GHDx
19683	Guinea	Guinea Demographic and Health Survey 2005	2005	Latitude/longitude	Macro International, Inc, National Statistics Directorate (Guinea). Guinea Demographic and Health Survey 2005. Fairfax, United States of America: ICF International.	GHDx
69761	Guinea	Guinea Demographic and Health Survey 2012	2012	Latitude/longitude	ICF Macro, Ministry of Health and Public Hygiene (Guinea), National Institute of Statistics (Guinea). Guinea Demographic and Health Survey 2012. Fairfax, United States of America: ICF International.	GHDx
303458	Guinea	Guinea Multiple Indicator Cluster Survey 2016	2016	Admin1	National Institute of Public Health (NPHI) (Guinea), National Institute of Statistics (Guinea), National Malaria Control Program (Guinea), United Nations Children's Fund (UNICEF). Guinea Multiple Indicator Cluster Survey 2016. New York, United States of America: United Nations Children's Fund (UNICEF), 2018.	GHDx
396957	Guinea	Guinea Demographic and Health Survey 2018	2018	Latitude/longitude	Ministry of Health (Guinea), Ministry of Planning and Economic Development (Guinea), National Institute of Statistics (Guinea). Guinea Demographic and Health Survey 2018. Fairfax, United States of America: ICF International, 2019.	GHDx

4808	Guinea-Bissau	Guinea-Bissau Multiple Indicator Cluster Survey 2000	2000	Admin1	Secretary State of Planning, National Institute of Statistics and Census (INEC), United Nations Children's Fund (UNICEF). Guinea-Bissau Multiple Indicator Cluster Survey 2000. New York, United States: United Nations Children's Fund (UNICEF).	GHDx
4818	Guinea-Bissau	Guinea-Bissau Multiple Indicator Cluster Survey 2006	2006	Admin1	United Nations Children's Fund (UNICEF), Government of Guinea-Bissau. Guinea-Bissau Multiple Indicator Cluster Survey 2006. New York, United States: United Nations Children's Fund (UNICEF).	GHDx
174049	Guinea-Bissau	Guinea-Bissau Multiple Indicator Cluster Survey 2014	2014	Admin1	National Statistics Institute (Guinea-Bissau), United Nations Children's Fund (UNICEF). Guinea-Bissau Multiple Indicator Cluster Survey 2014. New York, United States of America: United Nations Children's Fund (UNICEF), 2016.	GHDx
457894	Guinea-Bissau	Guinea-Bissau Multiple Indicator Cluster Survey 2018-2019	2018-2019	Admin1	National Statistics Institute (Guinea-Bissau), United Nations Children's Fund (UNICEF). Guinea-Bissau Multiple Indicator Cluster Survey 2018-2019. New York, United States of America: United Nations Children's Fund (UNICEF), 2020.	GHDx
20191	Liberia	Liberia Demographic and Health Survey 2006-2007	2006-2007	Latitude/longitude	Liberia Institute for Statistics and Geo-information Services (LISGIS), Macro International, Inc. Liberia Demographic and Health Survey 2006-2007. Fairfax, United States of America: ICF International.	GHDx
77385	Liberia	Liberia Demographic and Health Survey 2013	2013	Latitude/longitude	ICF International, Liberia Institute for Statistics and Geo-information Services (LISGIS), National AIDS and STI Control Program (NACP), Ministry of Health and Social Welfare (Liberia). Liberia Demographic and Health Survey 2013. Fairfax, United States of America: ICF International.	GHDx
286768	Liberia	Liberia Malaria Indicator Survey 2016	2016	Latitude/longitude	ICF International, Liberia Institute for Statistics and Geo-information Services (LISGIS), National Malaria Control Program (Liberia). Liberia Malaria Indicator Survey 2016. Fairfax, United States of America: ICF International, 2017.	GHDx
459845	Liberia	Liberia Demographic and Health Survey 2019-2020	2019-2020	Latitude/longitude	ICF International, Liberia Institute for Statistics and Geo-information Services (LISGIS), Ministry of Health and Social Welfare (Liberia). Liberia Demographic and Health Survey 2019-2020. 2021.	GHDx
20274	Mali	Mali Demographic and Health Survey 2006	2006	Latitude/longitude	Macro International, Inc, Ministry of Health (Mali), National Directorate of Statistics and Informatics (DNSI) (Mali). Mali Demographic and Health Survey 2006. Fairfax, United States of America: ICF International.	GHDx
77388	Mali	Mali Demographic and Health Survey 2012-2013	2012-2013	Latitude/longitude	ICF International, INFO-STAT (Mali), Ministry of Health (Mali), National Institute of Statistics (INSTAT) (Mali), Planning and Statistics Unit, Ministry of Health (Mali). Mali Demographic and Health Survey 2012-2013. Fairfax, United States of America: ICF International, 2014.	GHDx
248224	Mali	Mali Multiple Indicator Cluster Survey 2015	2015	Admin1	Ministry of Health (Mali), Ministry of Planning (Mali), National Institute of Statistics (INSTAT) (Mali), United Nations Children's Fund (UNICEF). Mali Multiple Indicator Cluster Survey 2015. New York, United States of America: United Nations Children's Fund (UNICEF), 2017.	GHDx

20315	Mali	Mali Demographic and Health Survey 2001	2001	Latitude/longitude	Macro International, Inc, National Directorate of Statistics and Informatics (DNSI) (Mali), Planning and Statistics Unit, Ministry of Health (Mali). Mali Demographic and Health Survey 2001. Fairfax, United States of America: ICF International.	GHDx
260407	Mali	Mali Agricultural Integrated Economic Survey 2014-2015	2014-2015	Admin1	Ministry of Rural Development (Mali), National Institute of Statistics (INSTAT) (Mali), World Bank. Mali Agricultural Integrated Economic Survey 2014-2015. Washington DC, United States of America: World Bank.	GHDx
398033	Mali	Mali Demographic and Health Survey 2018	2018	Latitude/longitude	ICF International, National Institute of Statistics (INSTAT) (Mali). Mali Demographic and Health Survey 2018. Fairfax, United States of America: ICF International, 2019.	GHDx
8115	Mauritania	Mauritania Multiple Indicator Cluster Survey 2007	2007	Admin3	National Office of Statistics (Mauritania), United Nations Children's Fund (UNICEF). Mauritania Multiple Indicator Cluster Survey 2007. New York, United States of America: United Nations Children's Fund (UNICEF).	GHDx
20322	Mauritania	Mauritania Demographic and Health Survey 2000-2001	2000-2001	Admin3	Macro International, Inc, National Office of Statistics (Mauritania). Mauritania Demographic and Health Survey 2000-2001. Fairfax, United States of America: ICF International.	GHDx
152783	Mauritania	Mauritania Multiple Indicator Cluster Survey 2011	2011	Admin3	National Office of Statistics (Mauritania), United Nations Children's Fund (UNICEF). Mauritania Multiple Indicator Cluster Survey 2011. New York, United States of America: United Nations Children's Fund (UNICEF), 2015.	GHDx
267343	Mauritania	Mauritania Multiple Indicator Cluster Survey 2015	2015	Admin1	National Office of Statistics (Mauritania), United Nations Children's Fund (UNICEF). Mauritania Multiple Indicator Cluster Survey 2015. New York, United States of America: United Nations Children's Fund (UNICEF), 2018.	GHDx
9439	Niger	Niger Multiple Indicator Cluster Survey 2000	2000	Admin2	Government of Niger, Macro International, Inc, United Nations Children's Fund (UNICEF). Niger Multiple Indicator Cluster Survey 2000. New York, United States of America: United Nations Children's Fund (UNICEF).	GHDx
20499	Niger	Niger Demographic and Health Survey 2006	2006	Admin1	Department of Statistics and National Accounts (Niger), Macro International, Inc. Niger Demographic and Health Survey 2006. Fairfax, United States of America: ICF International.	GHDx
74393	Niger	Niger Demographic and Health Survey 2012	2012	Latitude/longitude	ICF International, Ministry of Public Health (Niger), National Institute of Statistics (Niger). Niger Demographic and Health Survey 2012. Fairfax, United States of America: ICF International, 2019.	GHDx
26866	Sao Tome and Principe	Sao Tome and Principe Demographic and Health Survey 2008-2009	2008-2009	Admin2	ICF Macro, Ministry of Health (Sao Tome and Principe), National Institute of Statistics (Sao Tome and Principe). Sao Tome and Principe Demographic and Health Survey 2008-2009. Fairfax, United States of America: ICF International.	GHDx
27055	Sao Tome and Principe	Sao Tome and Principe Multiple Indicator Cluster Survey 2000	2000	Admin1	National Institute of Statistics (Sao Tome and Principe), United Nations Children's Fund (UNICEF). Sao Tome and Principe Multiple Indicator Cluster Survey 2000. New York, United States of America: United Nations Children's Fund (UNICEF).	GHDx

214640	Sao Tome and Principe	Sao Tome and Principe Multiple Indicator Cluster Survey 2014	2014	Admin1	Global Fund to Fight Aids Tuberculosis and Malaria (GFATM), ICF International, National Center for Endemic Diseases (CNE) (Sao Tome and Principe), National Institute of Statistics (Sao Tome and Principe), United Nations Children's Fund (UNICEF), United Nations Development Programme (UNDP). Sao Tome and Principe Multiple Indicator Cluster Survey 2014. New York, United States of America: United Nations Children's Fund (UNICEF), 2016.	GHDx
464540	Sao Tome and Principe	Sao Tome and Principe Multiple Indicator Cluster Survey 2019	2019	Admin2	National Institute of Statistics (Sao Tome and Principe), United Nations Children's Fund (UNICEF). Sao Tome and Principe Multiple Indicator Cluster Survey 2019	GHDx
26855	Senegal	Senegal Demographic and Health Survey 2005	2005	Latitude/longitude	Ministry of Health and Prevention (Senegal), Research Center for Human Development (Senegal). Senegal Demographic and Health Survey 2005. Fairfax, United States of America: ICF International.	GHDx
56063	Senegal	Senegal Demographic and Health Survey 2010-2011	2010-2011	Latitude/longitude	Center for Research in Human Development (CRDH), Cheikh Anta Diop University, Hospital Aristide Le Dantec, ICF Macro, National Agency of Statistics and Demography (Senegal). Senegal Demographic and Health Survey 2010-2011. Fairfax, United States of America: ICF International.	GHDx
111432	Senegal	Senegal Continuous Demographic and Health Survey 2012-2013	2012-2013	Latitude/longitude	ICF International, Ministry of Health and Social Action (Senegal), National Agency of Statistics and Demography (Senegal). Senegal Continuous Demographic and Health Survey 2012-2013. Fairfax, United States of America: ICF International.	GHDx
191270	Senegal	Senegal Continuous Demographic and Health Survey 2014	2014	Latitude/longitude	Cheikh Anta Diop University, ICF International, National Agency of Statistics and Demography (Senegal). Senegal Continuous Demographic and Health Survey 2014. Fairfax, United States of America: ICF International.	GHDx
218592	Senegal	Senegal Continuous Demographic and Health Survey 2015	2015	Latitude/longitude	Cheikh Anta Diop University, ICF International, National Agency of Statistics and Demography (Senegal). Senegal Continuous Demographic and Health Survey 2015. Fairfax, United States of America: ICF International, 2016.	GHDx
286772	Senegal	Senegal Continuous Demographic and Health Survey 2016	2016	Latitude/longitude	ICF International, Ministry of Health and Social Action (Senegal), National Agency of Statistics and Demography (Senegal). Senegal Continuous Demographic and Health Survey 2016. Fairfax, United States of America: ICF International, 2017.	GHDx
287639	Senegal	Senegal - Dakar Urban Multiple Indicator Cluster Survey 2015-2016	2015-2016	Admin1	National Agency of Statistics and Demography (Senegal), United Nations Children's Fund (UNICEF). Senegal - Dakar Urban Multiple Indicator Cluster Survey 2015-2016. New York, United States of America: United Nations Children's Fund (UNICEF), 2018.	GHDx
450419	Senegal	Senegal Continuous Demographic and Health Survey 2018	2018	Admin1	Directorate of Forecasting and Statistics, Ministry of the Economy, Finance and Planning (Senegal), ICF International, Ministry of Health and Social Action (Senegal), United States Agency for International Development	GHDx

					(USAID). Senegal Continuous Demographic and Health Survey 2018. Fairfax, United States of America: ICF International, 2020.	
460813	Senegal	Senegal Continuous Demographic and Health Survey 2019	2019	Latitude/longitude	ICF International, Ministry of Health and Social Action (Senegal), National Agency of Statistics and Demography (Senegal), United States Agency for International Development (USAID). Senegal Continuous Demographic and Health Survey 2019. Fairfax, United States of America: ICF International, 2020.	GHDx
353526	Senegal	Senegal Continuous Demographic and Health Survey 2017	2017	Latitude/longitude	ICF International, Ministry of Health and Social Action (Senegal), National Agency of Statistics and Demography (Senegal), Unit for the Fight Against Malnutrition (Senegal). Senegal Continuous Demographic and Health Survey 2017. Fairfax, United States of America: ICF International, 2018.	GHDx
11639	Sierra Leone	Sierra Leone Multiple Indicator Cluster Survey 2000	2000	Admin1	Central Statistics Office (Sierra Leone), United Nations Children's Fund (UNICEF). Sierra Leone Multiple Indicator Cluster Survey 2000. New York, United States of America: United Nations Children's Fund (UNICEF).	GHDx
11649	Sierra Leone	Sierra Leone Multiple Indicator Cluster Survey 2005	2005	Admin2	United Nations Children's Fund (UNICEF), Statistics Sierra Leone. Sierra Leone Multiple Indicator Cluster Survey 2005. New York, United States: United Nations Children's Fund (UNICEF).	GHDx
21258	Sierra Leone	Sierra Leone Demographic and Health Survey 2008	2008	Latitude/longitude	Macro International, Inc, Statistics Sierra Leone. Sierra Leone Demographic and Health Survey 2008. Fairfax, United States of America: ICF International.	GHDx
76700	Sierra Leone	Sierra Leone Multiple Indicator Cluster Survey 2010	2010	Admin2	Statistics Sierra Leone, United Nations Children's Fund (UNICEF). Sierra Leone Multiple Indicator Cluster Survey 2010. New York, United States of America: United Nations Children's Fund (UNICEF).	GHDx
131467	Sierra Leone	Sierra Leone Demographic and Health Survey 2013	2013	Latitude/longitude	ICF International, Ministry of Health and Sanitation (Sierra Leone), Statistics Sierra Leone. Sierra Leone Demographic and Health Survey 2013. Fairfax, United States of America: ICF International, 2014.	GHDx
218619	Sierra Leone	Sierra Leone Multiple Indicator Cluster Survey 2017	2017	Admin2	Statistics Sierra Leone, United Nations Children's Fund (UNICEF). Sierra Leone Multiple Indicator Cluster Survey 2017. New York, United States of America: United Nations Children's Fund (UNICEF), 2018.	GHDx
12896	Togo	Togo Multiple Indicator Cluster Survey 2006	2006	Precise place names	Directorate General of Statistics and National Accounting (Togo), United Nations Children's Fund (UNICEF). Togo Multiple Indicator Cluster Survey 2006. New York, United States: United Nations Children's Fund (UNICEF).	GHDx
40021	Togo	Togo Multiple Indicator Cluster Survey 2010	2010	Precise place names	Directorate General of Statistics and National Accounting (Togo), United Nations Children's Fund (UNICEF). Togo Multiple Indicator Cluster Survey 2010. New York, United States: United Nations Children's Fund (UNICEF).	GHDx
77515	Togo	Togo Demographic and Health Survey 2013-2014	2013-2014	Latitude/longitude	Directorate General of Statistics and National Accounts (Togo), ICF International, Ministry of Health (Togo), Ministry of Planning, Development and Zoning (Togo). Togo Demographic and Health Survey 2013-2014. Fairfax, United States of America: ICF International, 2015.	GHDx

429991	Togo	Togo Multiple Indicator Cluster Survey 2017	2017	Latitude/longitude	Directorate General of Statistics and National Accounting (Togo), United Nations Children's Fund (UNICEF). Togo Multiple Indicator Cluster Survey 2010. New York, United States: United Nations Children's Fund (UNICEF).	GHDx
393869	Togo	Incomplete immunization among children aged 12-23 months in Togo: a multilevel analysis of individual and contextual factors	2017	Admin1	Ekouevi DK, Gbeasor-Komlanvi FA, Yaya I, Zida-Compaore WI, Boko A, Sewu E, Lacle A, Ndibu N, Toke Y, Landoh DE. Incomplete immunization among children aged 12-23 months in Togo: a multilevel analysis of individual and contextual factors. BMC Public Health. 2018; 18(1): 952.	GHDx
12886	Togo	Togo Multiple Indicator Cluster Survey 2000	2000	Admin2	United Nations Children's Fund (UNICEF). Togo Multiple Indicator Cluster Survey 2000. New York, United States of America: United Nations Children's Fund (UNICEF).	GHDx

Supplementary Table 3: Survey sources excluded from analysis
Citations, series, country, and years provided for each excluded data set along with rationale for their exclusion. This table includes results of all countries in western sub-Saharan Africa, including Nigeria, from the GHDx, that were not included in final analysis.

GHDx ID	Country	Series	Year(s)	Birth Cohort Removed	Citation	Rationale for exclusion
79839	Benin	Benin Demographic and Health Survey 2011-2012	2011-2012	all	ICF International, National Institute of Statistics and Economic Analysis (INSAE) (Benin), National Program Against AIDS (PNLS) (Benin). Benin Demographic and Health Survey 2011-2012. Fairfax, United States of America: ICF International, 2014.	Estimates considered implausible. Survey estimates are inconsistent with admin estimates and estimates from other established survey series (Benin Demographic and Health Survey 2006). Survey removed from analysis.
79839	Benin	Benin Demographic and Health Survey 2011-2012	2011-2012	all	ICF International, National Institute of Statistics and Economic Analysis (INSAE) (Benin), National Program Against AIDS (PNLS) (Benin). Benin Demographic and Health Survey 2011-2012. Fairfax, United States of America: ICF International, 2014.	Estimates considered implausible. Survey estimates are inconsistent with admin estimates and estimates from other established survey series (Benin Demographic and Health Survey 2006). Survey removed from analysis.
104957	Benin	Benin Health Statistical Yearbook 2009	2009	all	Ministry of Health (Benin). Benin Health Statistical Yearbook 2009. Porto-Novo, Benin: Ministry of Health (Benin), 2010.	Estimates considered implausible. Survey estimates are systematically high compared to admin estimates and estimates from other established surveys series (Benin Demographic and Health Survey 2006, Benin Multiple Indicator Cluster Survey 2014). Survey removed from analysis.
104956	Benin	Benin Health Statistical Yearbook 2010	2010	all	Ministry of Health (Benin). Benin Health Statistical Yearbook 2010. Porto-Novo, Benin: Ministry of Health (Benin), 2011.	Estimates considered implausible. Survey estimates are systematically high compared to admin estimates and estimates from other established surveys series (Benin Demographic and Health Survey 2006, Benin Multiple Indicator Cluster Survey 2014). Survey removed from analysis.
104908	Benin	Benin Health Statistical Yearbook 2011	2011	all	Ministry of Health (Benin). Benin Health Statistical Yearbook 2011. Porto-Novo, Benin: Ministry of Health (Benin), 2012.	Estimates considered implausible. Survey estimates are systematically high compared to admin estimates and estimates from other established surveys series (Benin Demographic and Health Survey 2006,

						Benin Multiple Indicator Cluster Survey 2014). Survey removed from analysis.
21502	Burkina Faso	Burkina Faso World Health Survey 2002-2003	2002-2003	all	World Health Organization (WHO). Burkina Faso World Health Survey 2002-2003. Geneva, Switzerland: World Health Organization (WHO), 2005.	Estimates considered implausible. Survey estimates are inconsistent with admin estimates and estimates from other established survey series (Burkina Faso Demographic and Health Survey 2003). Survey removed from analysis.
244455	Cameroon	Cameroon Multiple Indicator Cluster Survey 2014	2014	select	Ministry of Public Health (Cameroon), National Institute of Statistics (Cameroon), United Nations Children's Fund (UNICEF). Cameroon Multiple Indicator Cluster Survey 2014. New York, United States of America: United Nations Children's Fund (UNICEF), 2017.	Survey estimates include implausibly high coverage rates of 100% for some age cohorts. Select age cohorts removed from analysis.
217919	Cape Verde	Cape Verde Survey on DTP3 and Measles Coverage 2009	2009	all	Cape Verde Survey on DTP3 and Measles Coverage 2009.	Survey estimates include implausibly high coverage rates of 100%. Survey removed from analysis.
217919	Cape Verde	Cape Verde Survey on DTP3 and Measles Coverage 2009	2009	all	Cape Verde Survey on DTP3 and Measles Coverage 2009.	Survey estimates include implausibly high coverage rates of 100%. Survey removed from analysis.
218611	Cote d'Ivoire	Côte d’Ivoire Multiple Indicator Cluster Survey 2016	2016	all	National Institute of Statistics (Côte d’Ivoire), United Nations Children’s Fund (UNICEF). Côte d’Ivoire Multiple Indicator Cluster Survey 2016. New York, United States of America: United Nations Children’s Fund (UNICEF), 2018.	Survey estimates include implausibly high coverage rates of 100%. Survey removed from analysis.
218611	Cote d'Ivoire	Côte d’Ivoire Multiple Indicator Cluster Survey 2016	2016	all	National Institute of Statistics (Côte d’Ivoire), United Nations Children's Fund (UNICEF). Côte d’Ivoire Multiple Indicator Cluster Survey 2016. New York, United States of America: United Nations Children’s Fund (UNICEF), 2018.	Survey estimates include implausibly high coverage rates of 100%. Survey removed from analysis.
21622	Ghana	Ghana World Health Survey 2003	2003	all	World Health Organization (WHO). Ghana World Health Survey 2003. Geneva, Switzerland: World Health Organization (WHO), 2005.	Estimates considered implausible. Survey estimates are inconsistent with admin estimates and estimates from other established survey series (Ghana Demographic and Health Survey 2003). Survey removed from analysis.

165101	Ghana	Ghana Living Standards Measurement Survey 2012-2013	2012-2013	all	Ghana Statistical Service, World Bank. Ghana Living Standards Measurement Survey 2012-2013. Accra, Ghana: Ghana Statistical Service.	Estimates considered implausible. Survey estimates are systematically low compared to admin estimates and estimates from other established surveys series (Ghana Multiple Indicator Cluster Survey 2011). Survey removed from analysis.
236205	Ghana	Ghana Socioeconomic Panel Survey 2009-2010	2009-2010	all	Economic Growth Center, Yale University, Institute of Statistical, Social and Economic Research, University of Ghana. Ghana Socioeconomic Panel Survey 2009-2010. Washington, DC, United States: World Bank.	DPT3 estimates are systematically low compared to admin estimates and estimates from other established surveys series (Ghana Multiple Indicator Cluster Survey 2011). DPT3 removed from analysis.
21622	Ghana	Ghana World Health Survey 2003	2003	all	World Health Organization (WHO). Ghana World Health Survey 2003. Geneva, Switzerland: World Health Organization (WHO), 2005.	Estimates considered implausible. Survey estimates are inconsistent with admin estimates and estimates from other established survey series (Ghana Demographic and Health Survey 2003). Survey removed from analysis.
165101	Ghana	Ghana Living Standards Measurement Survey 2012-2013	2012-2013	all	Ghana Statistical Service, World Bank. Ghana Living Standards Measurement Survey 2012-2013. Accra, Ghana: Ghana Statistical Service.	Estimates considered implausible. Survey estimates are systematically low compared to admin estimates and estimates from other established surveys series (Ghana Multiple Indicator Cluster Survey 2011). Survey removed from analysis.
236205	Ghana	Ghana Socioeconomic Panel Survey 2009-2010	2009-2010	all	Economic Growth Center, Yale University, Institute of Statistical, Social and Economic Research, University of Ghana. Ghana Socioeconomic Panel Survey 2009-2010. Washington, DC, United States: World Bank.	DPT3 estimates are systematically low compared to admin estimates and estimates from other established surveys series (Ghana Multiple Indicator Cluster Survey 2011). DPT3 removed from analysis.
151797	Nigeria	Nigeria General Household Survey 2012-2013	2012-2013	all	National Bureau of Statistics (Nigeria). Nigeria General Household Survey 2012-2013. Washington, DC, United States of America: World Bank.	DPT3 estimates are systematically high compared to admin estimates and estimates from other established surveys series (Nigeria Demographic and Health Survey 2013). DPT3 removed from analysis.
151802	Nigeria	Nigeria General Household Survey 2010-2011	2010-2011	all	National Bureau of Statistics (Nigeria). Nigeria General Household Survey 2010-2011. Abuja, Nigeria: National Bureau of Statistics (Nigeria).	Survey estimates are systematically high compared to admin estimates and estimates from other established survey series (Nigeria Demographic and Health Survey 2013). Survey removed from analysis.

222872	Nigeria	Nigeria Annual Report and Statement of Accounts 2002	1991-2002	all	Central Bank of Nigeria. Nigeria Annual Report and Statement of Accounts 2002. Abuja, Nigeria: Central Bank of Nigeria, 2003.	Estimates considered implausible. Survey estimates are systematically high compared to admin estimates and estimates from other established surveys series (Nigeria Multiple Indicator Cluster Survey 1999). Survey removed from analysis.
222875	Nigeria	Nigeria Annual Report and Statement of Accounts 2001	1991-2001	all	Central Bank of Nigeria. Nigeria Annual Report and Statement of Accounts 2001. Abuja, Nigeria: Central Bank of Nigeria, 2002.	Estimates considered implausible. Survey estimates are systematically high compared to admin estimates and estimates from other established surveys series (Nigeria Multiple Indicator Cluster Survey 1999). Survey removed from analysis.
151797	Nigeria	Nigeria General Household Survey 2012-2013	2012-2013	all	National Bureau of Statistics (Nigeria). Nigeria General Household Survey 2012-2013. Washington, DC, United States of America: World Bank.	DPT3 estimates are systematically high compared to admin estimates and estimates from other established surveys series (Nigeria Demographic and Health Survey 2013). DPT3 removed from analysis.
151802	Nigeria	Nigeria General Household Survey 2010-2011	2010-2011	all	National Bureau of Statistics (Nigeria). Nigeria General Household Survey 2010-2011. Abuja, Nigeria: National Bureau of Statistics (Nigeria).	Survey estimates are systematically high compared to admin estimates and estimates from other established survey series (Nigeria Demographic and Health Survey 2013). Survey removed from analysis.
222872	Nigeria	Nigeria Annual Report and Statement of Accounts 2002	1991-2002	all	Central Bank of Nigeria. Nigeria Annual Report and Statement of Accounts 2002. Abuja, Nigeria: Central Bank of Nigeria, 2003.	Estimates considered implausible. Survey estimates are systematically high compared to admin estimates and estimates from other established surveys series (Nigeria Multiple Indicator Cluster Survey 1999). Survey removed from analysis.
222875	Nigeria	Nigeria Annual Report and Statement of Accounts 2001	1991-2001	all	Central Bank of Nigeria. Nigeria Annual Report and Statement of Accounts 2001. Abuja, Nigeria: Central Bank of Nigeria, 2002.	Estimates considered implausible. Survey estimates are systematically high compared to admin estimates and estimates from other established surveys series (Nigeria Multiple Indicator Cluster Survey 1999). Survey removed from analysis.
50426	Nigeria	Nigeria Reproductive Health, Child Health, and Education Household, School,	2007	all	MEASURE Evaluation Project, Carolina Population Center, University of North Carolina, Center for Research, Evaluation, and Resource Development (CRERD), Center for Communication Programs, Bloomberg School of Public Health, Johns Hopkins, Creative Associates International, Constella Futures,	Estimates considered implausible. Survey estimates are inconsistent with admin estimates and estimates from other established survey series (Nigeria Multiple Indicator Cluster Survey 2007). Survey removed from analysis.

		and Health Facility Midline Surveys 2007			Adolescent Health and Information Project (Nigeria), Federation of Muslim Women's Associations of Nigeria (FOMWAN), Nigerian Medical Association, Management Sciences for Health (MSH), Civil Society Action Coalition on Education For All. Nigeria Reproductive Health, Child Health, and Education Household, School, and Health Facility Midline Surveys 2007. Chapel Hill, United States: MEASURE Evaluation Project, Carolina Population Center, University of North Carolina.	
24890	Nigeria	Nigeria General Household Survey 2007	2007	all	Central Bank of Nigeria, National Bureau of Statistics (Nigeria), Nigerian Communications Commission (NCC). Nigeria General Household Survey 2007. Abuja, Nigeria: National Bureau of Statistics (Nigeria).	Estimates considered implausible. Survey estimates are systematically low compared to admin estimates and estimates from other established surveys series (Nigeria Demographic and Health Survey 2008). Survey removed from analysis.
9522	Nigeria	Nigeria Core Welfare Indicators Questionnaire Survey 2006	2006	all	National Bureau of Statistics (Nigeria). Nigeria Core Welfare Indicators Questionnaire Survey 2006. Abuja, Nigeria: National Bureau of Statistics (Nigeria).	Estimates considered implausible. Survey estimates are systematically high compared to admin estimates and estimates from other established surveys series (Nigeria Demographic and Health Survey 2008). Survey removed from analysis.
50393	Nigeria	Nigeria Reproductive Health, Child Health, and Education Household, School, and Health Facility Baseline Surveys 2005	2005	all	MEASURE Evaluation Project, Carolina Population Center, University of North Carolina, Center for Research, Evaluation, and Resource Development (CRERD), Center for Communication Programs, Bloomberg School of Public Health, Johns Hopkins, Creative Associates International, Constella Futures, Adolescent Health and Information Project (Nigeria), Federation of Muslim Women's Associations of Nigeria (FOMWAN), Nigerian Medical Association, Management Sciences for Health (MSH), Civil Society Action Coalition on Education For All. Nigeria Reproductive Health, Child Health, and Education Household, School, and Health Facility Baseline Surveys 2005. Chapel Hill, United States: MEASURE Evaluation Project, Carolina Population Center, University of North Carolina.	Estimates considered implausible. Survey estimates are inconsistent with admin estimates and estimates from other established survey series (Nigeria Demographic and Health Survey 2008). Survey removed from analysis.
50426	Nigeria	Nigeria Reproductive	2007	all	MEASURE Evaluation Project, Carolina Population Center, University of North Carolina, Center for	Estimates considered implausible. Survey estimates are inconsistent with admin

		Health, Child Health, and Education Household, School, and Health Facility Midline Surveys 2007			Research, Evaluation, and Resource Development (CRERD), Center for Communication Programs, Bloomberg School of Public Health, Johns Hopkins, Creative Associates International, Constella Futures, Adolescent Health and Information Project (Nigeria), Federation of Muslim Women's Associations of Nigeria (FOMWAN), Nigerian Medical Association, Management Sciences for Health (MSH), Civil Society Action Coalition on Education For All. Nigeria Reproductive Health, Child Health, and Education Household, School, and Health Facility Midline Surveys 2007. Chapel Hill, United States: MEASURE Evaluation Project, Carolina Population Center, University of North Carolina.	estimates and estimates from other established survey series (Nigeria Demographic and Health Survey 2008). Survey removed from analysis.
50393	Nigeria	Nigeria Reproductive Health, Child Health, and Education Household, School, and Health Facility Baseline Surveys 2005	2005	all	MEASURE Evaluation Project, Carolina Population Center, University of North Carolina, Center for Research, Evaluation, and Resource Development (CRERD), Center for Communication Programs, Bloomberg School of Public Health, Johns Hopkins, Creative Associates International, Constella Futures, Adolescent Health and Information Project (Nigeria), Federation of Muslim Women's Associations of Nigeria (FOMWAN), Nigerian Medical Association, Management Sciences for Health (MSH), Civil Society Action Coalition on Education For All. Nigeria Reproductive Health, Child Health, and Education Household, School, and Health Facility Baseline Surveys 2005. Chapel Hill, United States: MEASURE Evaluation Project, Carolina Population Center, University of North Carolina.	Estimates considered implausible. Survey estimates are inconsistent with admin estimates and estimates from other established survey series (Nigeria Demographic and Health Survey 2008). Survey removed from analysis.
460813	Senegal	Senegal Continuous Demographic and Health Survey 2019	2019	select	ICF International, Ministry of Health and Social Action (Senegal), National Agency of Statistics and Demography (Senegal), United States Agency for International Development (USAID). Senegal Continuous Demographic and Health Survey 2019. Fairfax, United States of America: ICF International, 2020.	Survey estimates reflect catch-up vaccinations following 2018 health workers strike. Survey removed from analysis
425283	Sierra Leone	Sierra Leone Demographic and	2019	all	ICF International, Ministry of Health and Sanitation (Sierra Leone), Statistics Sierra Leone. Sierra Leone	Reported coverage inconsistent with unexplained decrease of 15 percent in the reported target population accompanied by

		Health Survey 2019			Demographic and Health Survey 2019. Fairfax, United States of America: ICF International, 2020.	a decrease in the reported number of administered doses. Survey removed from analysis

Supplementary Table 4: Final covariate set used for modelling
The final covariates used for modelling after selection using a variance inflation factor algorithm for selection.[2]

**Spatial covariates**	**National-level covariates**
Access to roads	Lag-distributed income
Distance from rivers or lakes	Prevalence of completion of the fourth antenatal care visit among pregnant women
Elevation	Mortality due to war and terrorism
Population	Bias-adjusted national-level administrative coverage
Urban or rural	
Irrigation	
Cloud cover percentage	
Frost day frequency	
Average daily minimum temperature	
Normalised difference vegetation index	
Multi-source Weighted-Ensemble Precipitation	
Tasseled cap brightness	
Nighttime land surface temperature	
Difference between daytime and nighttime land surface temperature	
Maternal education	

Supplementary References

1. ACLED. *ACLED* https://acleddata.com/.
2. Sbarra, A. N. *et al.* Mapping routine measles vaccination in low- and middle-income countries. *Nature* **589**, 415–419 (2021).
